# Measuring health related quality of life for dengue patients in Iquitos, Peru

**DOI:** 10.1371/journal.pntd.0008477

**Published:** 2020-07-28

**Authors:** William H. Elson, Amy R. Riley-Powell, Amy C. Morrison, Esther E. Gotlieb, Erik J. Groessl, Jhonny J. Cordova, J. Esther Rios, W. Lorena Quiroz, Alfonso S. Vizcarra, Robert C. Reiner, Christopher M. Barker, Gonzalo M. Vazquez-Prokopec, Thomas W. Scott, Alan L. Rothman, John P. Elder, Valerie A. Paz-Soldan

**Affiliations:** 1 Department of Entomology and Nematology, University of California Davis, Davis, California, United States of America; 2 Department of Global Community Health and Behavioral Sciences, Tulane School of Public Health and Tropical Medicine, New Orleans, Louisiana, United States of America; 3 Participation, Inclusion and Social Change, and Health and Nutrition Research Clusters, Institute of Development Studies at the University of Sussex, Brighton, United Kingdom; 4 Department of Pathology, Microbiology and Immunology, School of Veterinary Medicine University of California Davis, Davis, California, United States of America; 5 Virology and Emerging Infections Department, United States Naval Medical Research, Washington DC United States of America and Lima/Iquitos, Peru; 6 Department of Family Medicine and Public Health, University of California San Diego and VA San Diego Medical Center, San Diego, California, United States of America; 7 Department of Health Metrics Sciences, School of Medicine, University of Washington, Seattle, Washington, United States of America; 8 Department of Environmental Sciences, Emory University, Atlanta, Georgia, United States of America; 9 Institute for Immunology and Informatics and Department of Cell and Molecular Biology, University of Rhode Island, Providence, Rhode Island, United States of America; 10 Graduate School of Public Health, San Diego State University, San Diego, California, United States of America; University of Heidelberg, GERMANY

## Abstract

Previous studies measuring the health-related quality of life (HRQoL) of individuals with dengue focused on treatment seeking populations. However, the vast majority of global dengue cases are unlikely to be detected by health systems. Representative measurements of HRQoL should therefore include patients with disease not likely to trigger treatment-seeking behavior. This study based in Iquitos, Peru used the Quality of Wellbeing Scale-Self Administered, a survey that enquires about not only physical health, but also psychological health, self-care, mobility, and usual social activities, and rates HRQoL between 0 (death) and 1 (optimum function), to evaluate the impact of dengue on HRQoL. In order to enroll treatment and non treatment-seeking participants, three modalities of participant recruitment were used. In addition to clinic and community-based febrile surveillance, a contact-cluster methodology was also employed to identify infected individuals less likely to seek treatment. We measured changes in HRQoL and identified common areas of health impairment in 73 virologically confirmed dengue cases at 3 time points during the participant’s illness; the early-acute (days 0–6 post symptom onset), late-acute (days 7–20), and convalescent illness phases (days 21 +). Participants reported HRQoL related impairments at significantly higher frequency during the early-acute versus convalescent illness phase (Fisher’s exact: *P<0*.*01*). There was substantial heterogeneity in scores during each illness phase with median scores in the early-acute, late-acute and convalescent phases of 0.56 (IQR: 0.41–0.64), 0.70 (IQR: 0.57–0.94), and 1 (IQR: 0.80–1.00), respectively. In all illness phases participants recruited in clinics had on average the lowest HRQoL scores where as those recruited in the contact clusters had the highest. Only 1 individual who was recruited in the contact-clusters had no reduction in HRQoL score during their illness. These data illustrate that dengue should be considered as a disease that may have significant implications for not only physical health but also psychological health and social functioning. The impact of dengue on the HRQoL of non-treatment-seeking individuals, although lower than the impact among treatment-seeking individuals, is not necessarily trivial.

## Author summary

Dengue, a viral illness occurring following the bite of an infected mosquito, is common in tropical urban areas of the globe. Using a survey that enquires about not only physical health but also psychological health, self-care, mobility and usual social activities, this study assesses the overall impact of dengue on the well-being of those with the disease. Of the estimated 390 million annual infections, approximately 75 percent do not cause individuals to attend medical facilities, possibly because many infections do not result in significant symptoms or because access to healthcare is limited. Unlike previous studies, this study recruited those who would have been unlikely to seek healthcare as well as individuals who attended health facilities. This study adds to the growing body of evidence that healthcare professionals should regard dengue as a disease that affects not only physical, but also psychological health and aspects of social functioning. Furthermore, it suggests that those not seeking treatment may also have a substantial reduction in quality of life during their infection. Given the millions of dengue infected people who do not attend healthcare facilities annually this could amount to a very meaningful societal impact.

## Introduction

Dengue is a viral illness caused by one of four distinct serotypes of dengue virus (DENV) that are transmitted primarily by *Aedes aegypti* mosquitoes. It is a major global public health concern particularly in populated urban areas in the tropics [1,2]. Approximately 12 to 25% of the estimated 390 million annual global DENV infections are apparent, manifesting as a febrile illness with sufficient severity to alter an individual’s regular activity [3,4]. Symptomatic infection usually lasts 2 to 7 days and is characterized by fever accompanied by headache, fatigue, rash, and musculoskeletal pain [5]. Hospitalization rates for dengue in the Americas are reported as 11% with approximately 10,000 annual deaths globally [3,6].

Although literature describing the symptoms of dengue is abundant, a holistic understanding of the impact of dengue on an individual’s overall well-being also requires consideration of its effects on psychological health and social functioning (such as mobility and self-care), which are less well documented [7–10]. Herein, we will use the term health-related quality of life (HRQoL) to describe this multidimensional concept of ‘overall health’, although in the literature various largely interchangeable terms are used including quality of life and quality of well-being [11]. There are numerous non-disease-specific validated scales used to measure HRQoL, most commonly applied in the context of chronic disease [12].

Though limited in number, studies have shown that the reduction in HRQoL associated with dengue is a consequence not only of the physical symptoms, but also its impact on cognition, mood, interpersonal relationships and self-care [8–10]. In addition to improving our understanding of the holistic effects of a disease, studies of HRQoL may also be used to estimate disease burden. This approach uses the measured reduction in HRQoL during the course of an illness to determine the amount of healthy life lost during an illness episode. The values can in turn be used to estimate disability adjusted life years (DALYs), a measure of the years of healthy life lost due to a certain illness in a given population [13]. A recent systematic analysis by Zeng et al used empirical data from 6 studies to estimate the disease burden of a single, nonfatal dengue episode [14]. The authors concluded that previous measures may have underestimated the disease burden of non-fatal dengue with the average episode of dengue being responsible for the loss of 0.032 years of healthy life [3]. As these studies only recruited individuals who actively sought treatment, individuals with less severe symptoms and those who cannot access health facilities were excluded, an important limitation [9,14].

The prospective recruitment of non treatment-seeking individuals requires community-based strategies that enroll individuals prior to them attending health facilities. One such strategy is community febrile surveillance, where a cohort of individuals is monitored regularly and tested for dengue at the onset of symptoms. However, this approach excludes individuals that do not meet the threshold for inclusion (those with the mildest symptoms). To overcome this problem both geographic and contact-cluster strategies screen individuals at higher risk of infection prior to the onset of symptoms, based on their proximity (geographically or socially) to a symptomatic ‘index’ case [15,16]. This enables the potential recruitment of dengue positive individuals who may ultimately manifest any level of disease severity, including those who would not seek treatment. A number of cohort studies have successfully used location and contact-based cluster methods to recruit DENV infected individuals in the community who would be unlikely to seek healthcare, yet to our knowledge none have measured HRQoL in enrolled participants [15,16].

This study was part of a larger investigation based in the dengue endemic city of Iquitos, Peru that used clinic, community and contact-cluster based illness surveillance to recruit DENV-infected individuals from across the illness severity spectrum including those who would be less likely to seek treatment [17]. In this component of the study we applied a validated HRQoL survey to dengue participants addressing the following objectives: 1) describe the impact of dengue on physical and psychological health, as well as aspects of social wellbeing, 2) quantify the impact of dengue on HRQoL and describe how HRQoL changed over the course of dengue, and 3) determine the magnitude of the effect of each recruitment method on dengue-related changes in HRQoL.

## Methodology

### Ethics statement

The study protocol was approved by the Naval Medical Research Unit No. 6 (NAMRU-6) Institutional Review Board (IRB) (protocol #NAMRU6.2014.0028), in compliance with all applicable Federal regulations governing the protection of human subjects. IRB relying agreements were established between NAMRU-6, the University of California, Davis, Tulane University, Emory University and the University of California, San Diego. The protocol was reviewed and approved by the Loreto Regional Health Department, which oversees health research in Iquitos. Adults ≥ 18 years and parents or legal guardians of participants ≤17 years of age provided written informed consent or assent as appropriate.

### Field site

Data collection took place in the city of Iquitos, located in the Loreto Department of the Peruvian Amazon. The city is surrounded by three rivers; the Itaya, Nanay, and Amazon and is accessible only by plane or boat. According to the latest census, Iquitos has a population of approximately 400,000 with the principal industries being oil, fishing, lumber, tourism, and agriculture [17,18]. In 2016 more than 60% of the department population relied on government health insurance, which covers those living in poverty and extreme poverty [19]. Autochthonous transmission of all four DENV serotypes has been reported in Iquitos since 1990, with DENV2 currently being the predominant circulating serotype [18]. Zika virus was also introduced into Iquitos during late 2016 and circulated until the end of 2017 [20]. There are numerous publications covering approximately two decades of dengue-related epidemiological and entomological studies in Iquitos [18,21,22].

### Study design

We relied on 3 mechanisms for recruitment of study participants. The first two mechanisms were clinic-based passive surveillance [23] and community-based active surveillance [24]. Positive cases identified through either clinic or community-based surveillance were then used to initiate the third mechanism, active surveillance based on a contact-cluster design [16].

Clinic-based passive illness surveillance: Project nurses based at two Ministry of Health hospitals or seven health centers in and around Iquitos were responsible for enrolling clinic-based participants. Residents aged 5 years and older who presented with undifferentiated fever (≥ 38°C or reported use of antipyretics) for 5 days or less were offered a blood test to diagnose dengue using real-time polymerase chain reaction (RT-PCR). Individuals with virologically confirmed DENV infections were then invited to participate in prospective disease monitoring.

Community-based active illness surveillance: A team of nurses and phlebotomists recruited community-based participants in a cohort of greater than 25,000 residents from approximately 4,000 households in two neighborhoods in Iquitos. Houses were visited three times per week and residents were briefly asked about fever and other symptoms of dengue. Residents meeting the same criteria as clinic-based participants described above were invited to participate.

Contact-cluster active surveillance: A field team member experienced in dengue contact tracing visited DENV-positive participants recruited in either clinic-based or community-based surveillance. At this visit a retrospective movement survey was administered to DENV positive participants to determine locations visited in the previous 15 days [25]. Residential sites identified were visited and household members over 5 years were invited to provide an initial blood sample for RT-PCR testing; however, if another active case was detected in the initial samples then repeat samples were requested from all contacts at an interval of no less than 2 days. This methodology has been shown to facilitate the identification of asymptomatic or pre-symptomatic DENV infected individuals [16]. Positive individuals were invited to participate.

### Data collection

Participants were visited by the research team daily during the acute phase of illness until symptoms had resolved, and again 30 days after enrollment. Study physicians reviewed all DENV positive participants and could be contacted by field workers should subsequent medical advice be needed. The QWB-SA was one of a number of surveys administered to participants during their illness. The other surveys were a daily symptom survey, two surveys to capture participant movements, a sociodemographic survey and a survey assessing disease-related expenses.

The QWB-SA is a comprehensive measure of HRQoL that like the original QWB survey, is a preference-based measure of HRQoL that produces a single, summary score ranging from 0 (dead) to 1 (optimum function) and allows for the calculation of Quality-Adjusted Life Years (QALYs) [26]. Symptoms and functional limitations that are endorsed as present are multiplied by preference weights obtained in a large community sample [26]. The survey includes 8 principal sections, each with a varying number of questions (S1 Table and S1 Text). Sections 1 and 2 (total 43 questions) address physical symptoms. Section 3 (14 questions) addresses mental health symptoms. Section 4 is a single open-ended question enquiring about any other symptoms not already reported. Section 5 (2 questions) addresses whether help was needed with self-care. Section 6 (3 questions) addresses mobility. Section 7 (8 questions) considers physical functioning. Section 8 (3 questions) addresses usual social activity.

The QWB-SA has been used with a variety of chronic diseases, such as musculoskeletal and mental health disorders and epilepsy [23]. Although the application of this tool to dengue (an acute infectious disease) is novel, the QWB-SA was selected because it addresses common dengue symptoms and its questions focus on the three days preceding survey completion, which is an appropriate temporal scale for the course of dengue symptoms. The survey has substantially more questions than other HRQoL surveys (EuroQol and WHOQOL-BREF) previously used in dengue studies, and therefore we reasoned that its broader question coverage might help to capture subtle changes in HRQoL [8,26–28]. We created a digital version of the Spanish QWB-SA survey using CommCare, an open source, cloud-based platform that provides a framework for developing mobile data collection systems [29]. This tool was piloted by our research team, and a small number of linguistic modifications specific to Iquitos were made to the questions. The survey was then administered by experienced local field workers using a digital mobile device on day 3 and 7 post diagnosis and at day 30 to serve as a proxy for baseline HRQoL.

Completed surveys were uploaded to the CommCare Server and reviewed for data entry errors by senior project members. If necessary, data corrections were made using the CommCare HQ website data correction facility. Final versions of the completed surveys were then imported into a PostgreSQL database and anonymized survey data were sent to the University of California-San Diego where the QWB-SA algorithm was applied to each survey to generate the HRQoL score [26].

Forms were assigned a ‘day of illness’ (DOI), which indexes surveys by the number of days post symptom onset that they were acquired. The DOI was calculated by subtracting the date of symptom onset from the date one day prior to survey administration. DOI 0, therefore, represents the day symptoms started and, because contacts could be diagnosed prior to symptom onset, the DOI could be a negative value. Because the questions in the QWB-SA refer to the three days prior to the interview day, we regarded the survey as having referred to the DOI corresponding to the day before the interview. Surveys were then grouped into 1 of 3 illness phases: 1) surveys completed up to and including DOI 6 were assigned to the ‘early-acute phase’, 2) surveys from DOI 7 to 20 were assigned to the ‘late-acute phase’ and 3) surveys completed after DOI 20 were assigned to the ‘convalescent phase’ of illness.

We present results of individual questions from the QWB-SA in the following 5 groups; Physical symptoms (Sections 1 and 2), psychological symptoms (Section 3), self-care (Section 5), mobility and physical functioning (Sections 6 and 7) and usual social activity (Section 8). Section 1 and 2 have been combined as they both represent physical symptoms, Sections 6 and 7 are combined as these both relate strongly to mobility. For display purposes we used abbreviated versions of the questions and where necessary clarify the exact nature of the question in the text. See S1 Table for details of grouping and abbreviations.

All statistical analyses were carried out in R version 3.5.1 [30]. Basic descriptive statistics were calculated for baseline characteristics using means and standard deviations if variables were normally distributed, or medians and interquartile ranges for non-normally distributed variables. To assess for differences in responses between illness phases, the proportion of individuals responding affirmatively to all questions was calculated for each of the three illness phases. Comparisons of proportions were made using Fisher’s exact tests between illness phases. Due to the large number of comparisons a Bonferroni correction was applied to the alpha value transforming alpha levels of 0.05 and 0.01 to 0.0007 and 0.0001, respectively. As HRQoL scores were non-normally distributed, to analyze differences in HRQoL scores between illness phases and recruitment methodology we carried out Wilcoxon rank-sum tests for pairwise comparisons and Kruskal-Wallis tests for comparisons of more than two groups.

To further quantify changes in HRQoL over time and to evaluate the effects of important independent variables on HRQoL score, we considered a mixed-effects regression model using HRQoL score as the dependent variable. Independent variables in this model included the following fixed effects: (1) DOI, to measure the effect of the time course of illness; (2) participant recruitment method, to assess how the three different recruitment methods may sample from intrinsically different populations, with the expectation that those recruited by community-based active illness surveillance and contact-cluster active surveillance would have higher HRQoL scores than those recruited in the clinic-based surveillance; (3) Sex, as differing roles within in the household might affect HRQoL and could have important social implications, and (4) Age, as a continuous variable, as evidence suggests that the likelihood of symptomatic disease is affected by age [31]. We also included a random intercept by participant to account for repeated measures by person. As the HRQoL score (dependent variable) could take on values only between 0 and 1 we transformed the variable with a logit link function after applying a small adjustment to move values away from both 0 and 1 to prevent the occurrence of infinite values following transformation, this is called a logit normal regression. We then created a linear mixed effects regression model using the lmer function from the ‘lme4’ and ‘lmerTest’ packages in R [32]. Finally, we tested the significance of the random effect by participant by comparing models with and without the random effect using the likelihood ratio test. Analogously to an odds ratio for a logistic regression we report all results in terms of a “score ratio” (SR) which here is the score divided by one minus the score. Although not the primary purpose of this model we then used the model predicted scores to calculate the disease burden of an acute dengue episode in order to make comparisons with other similar reports in the literature. To do this we calculated the model predicted loss of HRQoL for the first 20 days of illness by subtracting predicted HRQoL scores for each day from 1 and adding these values together. This number represents the estimated number of healthy days lost to dengue in the first 20 days of illness. To complete the calculation, we divided this number by 365.25 to convert the number of lost days to the amount of lost years. We did this for each of the three recruitment methods.

## Results

### Subject characteristics

Table 1 summarizes baseline characteristics of the study participants (See S2 Table that summarizes data from only individuals who completed a survey in each illness phase). There were 73 DENV-positive participants contributing a total of 195 QWB-SA surveys. Of these 73 participants, 12 were recruited in clinics or hospitals by passive surveillance, 42 in the community-cohort by active surveillance and 19 in contact clusters. A total of 7 participants were hospitalized (at least one night spent as an inpatient) of which 1 was recruited by clinic-based surveillance, 5 by community-based surveillance, and 1 by contact-cluster surveillance. Fifty-six of the 73 participants completed 3 QWB-SA surveys, 10 completed 2 surveys and 7 completed 1 survey. Fifty of the 56 participants who completed 3 surveys completed a single survey in each illness phase. Of all participants recruited in the clinics, only 50% (6) went on to complete 3 surveys, compared to 81% (34) of those recruited in the community and 84% (16) recruited in the contact-clusters. Pairwise comparisons between these percentages were not statistically significant (Fisher’s exact test p>0.05).

**Table 1:** Participant characteristics.

Characteristic	All	Clinic	Community	Cluster
Number of participants	73	12	42	19
Number of surveys	195	27	115	53
Sex (%)				
Male	37 (51)	4 (33)	24 (57)	9 (47)
Female	36 (49)	8 (67)	18 (43)	10 (53)
Median age (IQR)	17 (13–27)	25 (19–39)	16 (13–18)	15 (11–33.5)
Median symptom duration in days (IQR)*	2 (1–3)	2.5 (1–3)	3 (1–4)	1 (0–1)
Hospitalized (%)	7 (10)	1 (8)	5 (12)	1 (5)
Serotype (%)				
DENV2	70 (96)	11 (92)	41 (98)	18 (95)
DENV3	3 (4)	1 (8)	1 (2)	1 (5)

*Median number of days of symptoms a participant had already experienced on the day the diagnostic blood sample was taken. S2 Table includes a version that contains data from individuals who completed a survey in each illness phase.

The age and sex distribution of those recruited from the community and contact-clusters was similar. The percentage of women recruited from clinic surveillance was non-significantly greater than from both the community surveillance (Fisher’s exact test p = 0.20) and the contact-clusters (Fisher’s exact test p = 0.48). The age of those recruited from the clinics was significantly greater than from community (Wilcoxon test p<0.01) and non-significantly greater than from contact-clusters (Wilcoxon test p = 0.08). Participants from the clinics and community had experienced a similar number of days of symptoms at recruitment, differences were small and non-significant (Wilcoxon test p = 0.73). However, participants from the contact-clusters were recruited after significantly less days of symptoms compared to both clinic participants (Wilcoxon test p<0.01) and community participants (Wilcoxon test p<0.01).

### Responses by illness phase

**Fig 1:**
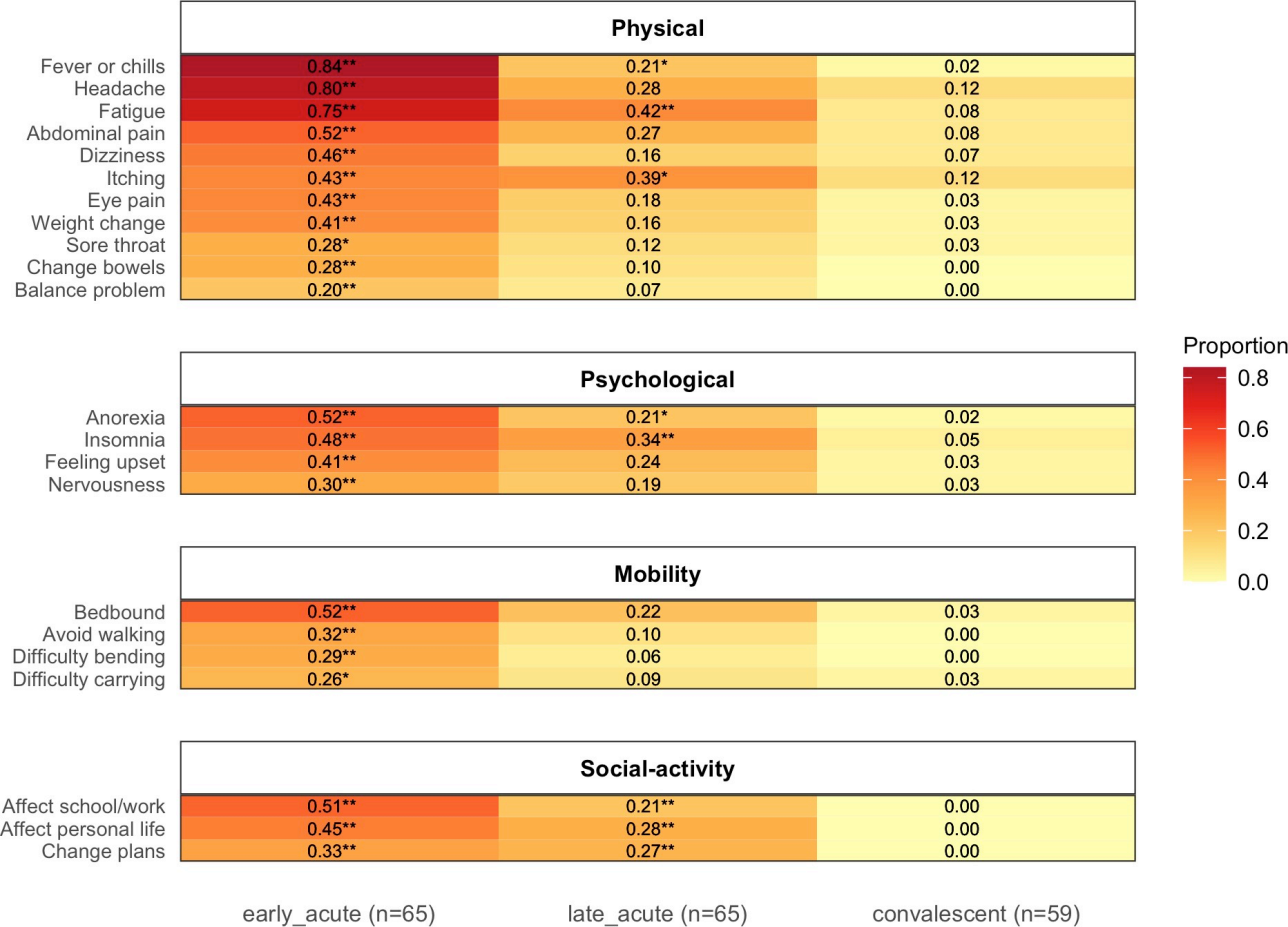
**Heatmap: Proportion of participants reporting symptoms or effects by illness phase** (left column: early-acute, middle column: late-acute, right column: convalescent phase). The proportion is represented by the redness of the cell and the actual proportion is shown by the number in each cell. * Fisher’s exact test: *P<0*.*05*, ** Fisher’s exact test: *P<0*.*01*. This includes only symptoms that were reported in significantly higher frequency in the early-acute or late-acute versus the convalescent phase of illness.

Early-acute versus convalescent phase: A significantly greater proportion of affirmative responses were recorded in 22 questions in the early-acute versus the convalescent illness phase (Fisher’s exact: *P<0*.*05*). Eleven of these were physical symptoms, including fever, headache and fatigue; 4 were psychological symptoms, anorexia, insomnia, feeling upset and nervousness; 4 related to mobility, including being bedbound, avoiding walking and difficulty bending and carrying items; 3 related to social activity and included an effect on school/work, an effect on personal life and a need to change plans for health reasons (Fig 1). All of these 22 responses showed a declining trend across the 3 illness phases, illustrated in Fig 1 by the color gradient from left to right. Heatmaps that include all questions by each recruitment mode can be seen in S1–S4 Figs. A paired analysis of individuals who completed 1 survey in each illness phase revealed very similar patterns (S5 Fig).

Late-acute versus convalescent phase: Affirmative responses that continued to be reported in significantly higher frequency in the late-acute compared with the convalescent phase (Fisher’s exact: *P<0*.*05*) included fever, insomnia, an effect on school/work and an effect on personal life. Notably, the only response reported in significantly higher frequency in the late-acute versus convalescent phase that was not also significantly elevated in the early-acute phase was being hospitalized (spending any part of the day or night as a patient in a hospital, nursing home, or rehabilitation center). The most frequent responses reported in the convalescent phase of illness were headache (7/59), itching (7/59), fatigue (5/59), abdominal pain (including upset stomach, abdominal pain, nausea, heartburn, or vomiting) (5/59) and anxiety (5/59). The paired analysis revealed that only an effect on personal life was reported in significantly higher frequency in the late-acute versus convalescent phase (S5 Fig). S3 Table also shows a comparison between the early-acute and late-acute illness phases.

### HRQoL score by illness phase (Table 2 and Fig 2)

The HRQoL scores were lowest in the early-acute phase of illness and showed improvement over the 3 illness phases with median scores in the early-acute, late-acute and convalescent phases of 0.56, 0.70 and 1.00, respectively. This trend was maintained in a paired analysis of the 50 participants who provided QWB-SA surveys in all 3 illness phases with median scores in the acute, early-acute, chronic phases of 0.52, 0.75 and 1.00, respectively (S4 Table, S6 Fig). In both the non-paired and paired analyses, the differences between early-acute and convalescent scores and between late-acute and convalescent scores were significant (Wilcoxon: *P<0*.*01*). Of the 50 individuals who provided scores in all 3 illness phases there was substantial heterogeneity in the differential between a participant’s maximum and minimum scores, with a mean difference of 0.42 (SD 0.137). Of these 50 participants all had maximum scores of greater than 0.6, 40 had maximum scores greater than 0.8 and 34 had maximum scores of 1, implying that the majority of individuals had good baseline HRQoL. The median minimum HRQoL scores in these 50 participants was 0.49 (IQR 0.41–0.6). The trend of improving HRQoL scores was maintained when data was grouped by both sex and age. Although the numbers of individuals within each of these subgroups were small the differences were statistically significant (Kruskal-Wallis *P<0*.*05*), except for females in the 18 to 53-year age group.

**Fig 2:**
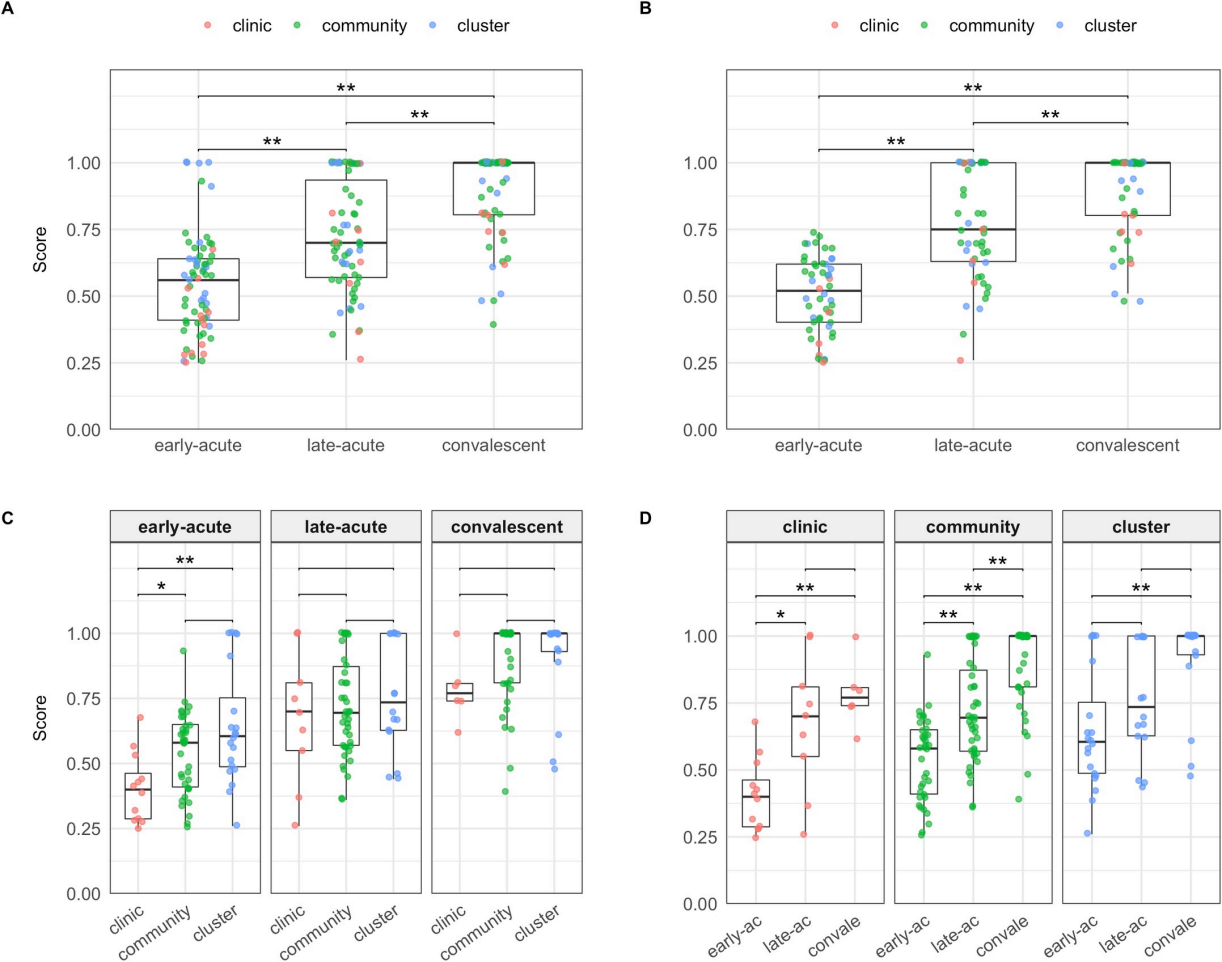
HRQoL score by illness phase Box and whisker plots demonstrate the distribution of HRQoL scores in each illness phase, dark horizontal line = median, upper limit of box = 75^th^ percentile, lower limit box = 25^th^ percentile, upper whisker extends to the largest value < = 1.5 multiplied x IQR, lower whisker extends to the smallest value > = 1.5 x IQR. Red dots represent individual scores. They are partially transparent and therefore appear darker where multiple points overlay each other. **A**: Un-paired analysis of all participants regardless of recruitment mode or the number of surveys completed, * Wilcoxon: *P<0*.*05*, ** Wilcoxon: *P<0*.*01*. **B**: Paired analysis of only participants who completed a single survey in each illness phase regardless of recruitment * Wilcoxon: *P<0*.*05*, ** Wilcoxon: *P<0*.*01*. **C:** Un-paired analysis comparing recruitment modes within each illness phase * Wilcoxon: *P<0*.*05*, ** Wilcoxon: *P<0*.*01*. **D**: Un-paired analysis comparing illness phases within each recruitment mode * Wilcoxon: *P<0*.*05*, ** Wilcoxon: *P<0*.*01*. Paired versions of C & D can be seen in S6 Fig.

**Table 2:** HRQoL Scores by illness phase and recruitment mode.

recruitment	phase	forms	participants	median score (IQR*)	range
All	Early-acute	69	65	0.56 (0.41–0.64)	0.25–1.00
	Late-acute	67	65	0.70 (0.57–0.94)	0.26–1.00
	Convalescent	59	59	1.00 (0.80–1.00)	0.39–1.00
Clinic	Early-acute	12	12	0.40 (0.29–0.46)	0.25–0.68
	Late-acute	9	9	0.70 (0.55–0.81)	0.26–1.00
	Convalescent	6	6	0.77 (0.74–0.81)	0.62–1.00
Community	Early-acute	37	36	0.58 (0.41–0.65)	0.26–0.93
	Late-acute	42	40	0.70 (0.57–0.87)	0.36–1.00
	Convalescent	36	36	1.00 (0.81–1.00)	0.39–1.00
Contact	Early-acute	20	17	0.60 (0.49–0.75)	0.26–1.00
	Late-acute	16	16	0.74 (0.63–1.00)	0.44–1.00
	Convalescent	17	17	1.00 (0.93–1.00)	0.48–1.00

*IQR: Interquartile range

### Multiple regression analysis (Table 3 and Fig 3)

The likelihood ratio test (LRT) comparing models with and without a random effect by participant found the random effect to non-significantly affect the model performance (*P = 0*.*138*); we therefore selected the model without the random effect. Although it could be argued due to the repeated measures by participant that selecting the model with a random effect by participant would be more appropriate regardless of the results of the LRT, the model coefficients are very similar and thus we chose the simpler of the two models.

**Fig 3:**
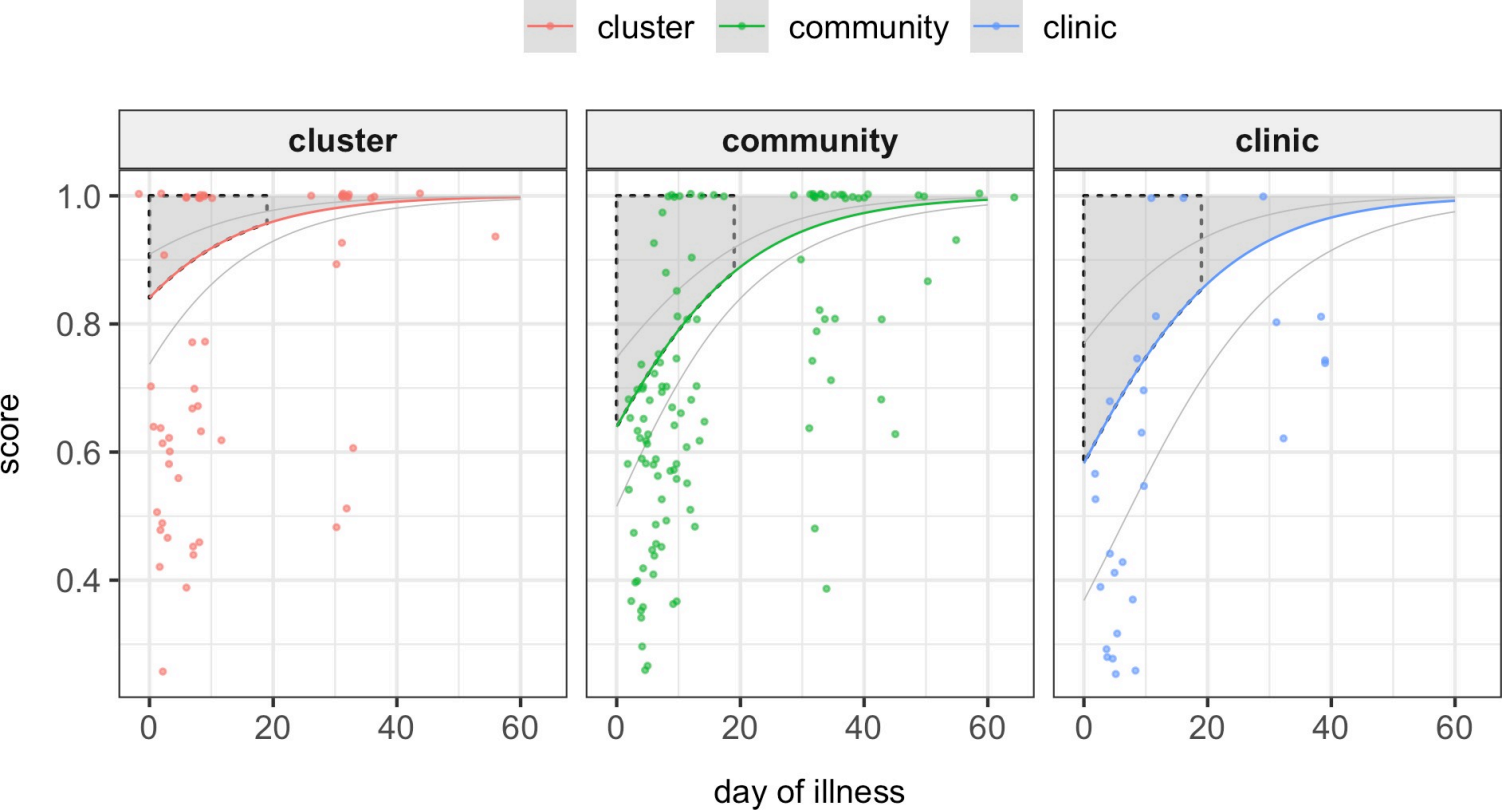
HRQoL by day of illness and recruitment modality Multiple regression analysis predicted HRQoL scores (y-axis) by day of illness (x-axis) and recruitment modality (individual panels). Individual points represent actual measured HRQoL scores. Thick colored lines represent model predicted scores and faint lines represent +/-2 x standard error. As the model included age as a continuous variable, we graphed predicted scores for the median age of 16 years and as the differences in sexes was negligible and non-significant, we only represent males. Shaded areas represent predicted healthy life lost due to a dengue episode. Black dashed lines enclose the proportion of healthy life lost within the first 20 days of illness.

**Table 3:** Model comparison.

Independent variables	Fixed effects model ^e^	Mixed effects model
	SR (95% CI)	SR (95% CI)
(Intercept)^a^	9.54 (4.54–20.07)	9.41 (4.24–20.93)
Day of illness	1.08 (1.06–1.10)	1.08 (1.06–1.10)
Recruitment: community-based^b^	0.34 (0.18–0.62)	0.33 (0.17–0.65)
Recruitment: clinic-based^b^	0.26 (0.11–0.63)	0.26 (0.10–0.66)
Age	0.96 (0.94–0.98)	0.96 (0.94–0.98)
Sex: Male^c^	1.09 (0.65–1.85)	1.09 (0.62–1.94)
Likelihood ratio test^d^	${Χ}_{1}^{2}=2.198$ *P = 0*.*138*	

**a:** Intercept Score ratio when all independent variables set to zero or (default if categorical) i.e. Day of illness = 0, Recruitment = contact-cluster, Age = 0 and Sex = female

**b:** Default = contact-cluster.

**c:** Default = female.

**d:** Likelihood ratio test comparing model performance between the 2 models. P-value of 0.138 suggests there is no significant difference between model performance.

^e^ Selected model. Model fixed effects score ratios (SR) and 95% confidence intervals (CI).

In our final model, day of illness was found to have a significant positive association with HRQoL (SR:1.08/day, CI:1.06–1.10). When compared to the contact-cluster modality of capture both community-based (SR:0.34, CI:0.18–0.62) and clinic-based (SR:0.26, CI:0.11–0.63) surveillance were found to have significant negative association with HRQoL. Age had a significant negative association with HRQoL score (SR:0.96/year, CI:0.94–0.98). Male sex had a non-significant positive association with HRQoL (SR:1.09, CI:0.65–1.85).

The predicted burden (years of healthy life lost) in the first 20 days of a single episode of dengue in a male of age 16 years recruited in the clinic, community and clusters was 0.015 (95% CI: 0.008–0.024), 0.012 (0.009–0.017) and 0.005 (0.003–0.008), respectively. These values are graphically represented by the shaded areas bound by the black dashed line in Fig 3.

## Discussion

In this study we describe the impact of dengue on the HRQoL of infected individuals. We have shown that the impairment of HRQoL in dengue is a function not only of its impact on physical, but also psychological health and aspects of social well-being. These results are consistent with three previous HRQoL studies in treatment-seeking dengue participants in Malaysia, Brazil and Vietnam [8–10]. During the acute phase of illness, typical physical symptoms of dengue were accompanied by nervousness and feelings of upset. The disease similarly had a number of effects on mobility and both personal and professional social activity that persisted into the late-acute phase of the illness. The 4 most common symptoms recorded in the convalescent phase (headache, itching, fatigue and anxiety) are similar to those in previous reports of persistent symptoms in dengue [33,34]. This information is of value to healthcare professionals managing dengue as it reinforces the importance of providing a holistic clinical assessment and draws attention to the potential impact of the disease beyond the febrile phase of the illness. By directly enquiring about symptoms of anxiety and the social consequences of dengue such as a reduced earning capacity, healthcare professionals could be prompted to direct patients to additional support such as mental health services. These lessons can also be applied to the population level ensuring that the appropriate resources, particularly in an outbreak scenario, are available to tackle the varied impacts of this disease.

We quantified the impact of dengue on HRQoL over the course of the illness showing that the HRQoL score is lowest during the acute illness phase and that there is a marked improvement in scores over the course of dengue. Although this pattern was the same for all recruitment modes, we showed that the magnitude of the reduction in HRQoL was associated with recruitment modality, where those recruited in the clinics tended to see the greatest reduction in HRQoL and those in the contact-clusters the least (Figs 2 and 3). The clinic recruited group was also notable for its older age than the other recruitment groups. Older age could potentially lead to lower HRQoL due to concurrent co-morbidities (we did not record these in this study). However, when adjusting for age using our multiple regression analysis, we show that clinic recruitment was still associated with a lower HRQoL score indicating that other factors are also involved. Overall these findings suggest that, as expected, those who seek treatment tend to be most adversely affected by dengue.

There was marked variation in the HRQoL scores in our entire study population and in the acute illness phase the range of scores was greatest in those recruited in the contact-clusters (Fig 2) where one individual had no reduction in HRQoL and one individual was ultimately hospitalized. This variation of HRQoL scores suggests that we sampled from a more heterogenous population than previous studies, with treatment and non treatment-seeking individuals included. We cannot label all participants as treatment or non treatment-seeking because the study intervention may have altered the behavior of those recruited in the community and contact-clusters as advice given to participants by study staff could have affected whether individuals attend a medical facility or not. It is tempting to assume that those with least impact on HRQoL represent those who would not have sought treatment. Whilst this is almost certainly true for the one individual who showed no reduced HRQoL during his illness, individuals experiencing a substantial reduction in HRQoL may not attend health facilities due to barriers to healthcare access such as financial factors and proximity [35]. We recruited only a single DENV positive individual who experienced no impairment in HRQoL during their infection despite it being stated that approximately 75% of DENV infections are inapparent. This discrepancy may indicate that the QWB-SA tool was sensitive enough to detect even small reductions in HRQoL that may not be detected by other measures of disease impact. However, an alternative interpretation is that the morbidity amongst individuals with infections regarded as inapparent is less trivial than assumed [4,9]. Whilst our data do not confirm this hypothesis, further support comes from cluster studies in children based in Thailand that reveal very few children reported no symptoms [36].

Another way to assess the impact of dengue on HRQoL is through the estimation of a disease burden metric such as DALYs. Using empirical data from 6 studies, Zeng et al, estimated that the first 20 days of a single episode of dengue are responsible for the loss of 0.012 years of healthy life [14]. Although the prediction of HRQoL scores was not the primary purpose of our multiple regression analysis using our model-predicted scores for males of the median age (16yrs) we calculated estimated DALYs for the first 20 days of a dengue episode for clinic, community and contact-cluster recruited participants. Whilst DALYs for clinic and community recruited participants were similar to the results of Zeng et al (0.015, 0.012), the contact-cluster group showed a substantially smaller DALY (0.005), again highlighting a greater disease heterogeneity in our entire study population and the presence of cases with a relatively smaller impact on HRQoL.

We would expect the impact of dengue on HRQoL scores in our study population to be less than in studies of only treatment-seeking populations. However, comparison of HRQoL scores between studies is complicated by methodological differences and differing study populations. The Malaysian and Brazilian studies used a visual analog scale (VAS) from the EuroQol survey that asks participants to place a mark on a ruler from 0 (worst health imaginable) to 100 (best health imaginable) indicating how they feel on that day [8,9,28]. In contrast the Vietnamese study used the EuroQol’s five-dimensional survey that enquires about each of the following; mobility, self–care, usual activities, pain/discomfort, and anxiety/depression [10]. A validated algorithm is then used to convert these responses into a HRQoL score [28]. Direct comparisons of the scores is further complicated by the differing timings of the data collection. The Malaysian study gathered data at 2 interviews asking participants to recall the impact of the disease on days that they were not interviewed for approximately 20 days. The Brazilian study took a similar approach but only performed a single interview at 15 days after symptom onset. The Vietnamese study does not clearly state when the interview occurred in relation to the disease onset.

The intention of using 3 recruitment modalities was to recruit a broader range of subjects. Although our results show that this was the case it is important to point out that each recruitment mode was subject to different selection biases. Clinic surveillance selects only those participants who can access a medical facility and those with symptoms severe enough to prompt treatment-seeking behavior. Thus, it is likely that those with milder symptoms will be under represented in this group. Community surveillance does enable recruitment of those who might not have access to healthcare facilities, but still only selects those who develop symptoms that meet the surveillance criteria. Contact-clusters overcome this problem by screening people whether or not they have specific symptoms. Nonetheless, participants in this group are selected based on their proximity to a symptomatic index case potentially meaning they too are more likely to develop symptoms due to, for example those in the same family may have similar immunological response to the illness due to genetic homogeneity. Whilst neither our total sample nor any of the three individual recruitment groups are likely to represent is to be representative of the 390 million dengue cases occurring globally, we have sampled participants with a broader spectrum of illness severity than other studies of HRQoL in dengue. We have shown that the findings from studies in treatment-seeking individuals should not be extrapolated to non treatment-seeking population.

We acknowledge a number of limitations in this study. We did not collect true baseline HRQoL data and although the majority of our participants (34/50) reported near perfect HRQoL at the follow-up interview, for those that did not we are unsure if the residual issues represented the persistent effects of dengue or baseline health issues. Future work would aim to take this into account and administer a retrospective pre-illness survey for comparison. Finally, compared to other HRQoL studies in dengue the sample size here is relatively small; 73 versus 372 in the Brazilian study and 207 in the Malaysian study [8,9]. This may have led to insufficient power to detect significant differences in some of the statistical comparisons. One of the principal reasons for reduced participant numbers was the introduction of Zika virus midway through this study during late 2016 at which time DENV transmission largely ceased [20]. However, unlike most publications we present data from multiple time points per participant enabling analysis of the trends of HRQoL scores over time. Although other publications did collect multiple data points these were collected retrospectively at a maximum of 2 interviews with the potential for substantial recall bias [8,9].

In summary, through the novel application of the QWB-SA we add to the growing body of evidence that dengue should be considered as a disease that may have significant implications for not only physical health but also psychological health and social functioning and that these health impairments may extend beyond the febrile period. These data illustrate the significant impact of dengue on HRQoL in a population that includes non-treatment seeking individuals, a previously understudied group. The findings suggest that measurements of HRQoL in treatment-seeking populations should not be extrapolated to non treatment-seeking groups and that the impact of dengue on the HRQoL of non-treatment seeking individuals, although lower than the impact among treatment seeking individuals, is not necessarily trivial. Further work to expand and improve the recruitment of non-treatment seeking individuals would enable a clearer distinction between these groups and contribute to a better understanding of impact of dengue on HRQoL in these groups.

## Supporting information

S1 Text Original QWB-SA survey in Spanish and English. (PDF)

S1 Table Survey questions and question groupings. (PDF)

S2 Table Participant characteristics for subset of individuals who completed a survey in each study phase. (PDF)

S3 Table Frequency (%) of all physical and psychological symptoms and social limitations by phase of illness. (PDF)

S4 Table HRQoL Scores by illness phase and recruitment mode only for subset of individuals who completed a survey in each study phase. (PDF)

S1 Fig Heatmaps: Proportion of participants reporting symptoms or effects by illness phase (includes all questions). All participants from all recruitment modes regardless of the number of forms completed.
(PDF)

S2 Fig Heatmaps: Proportion of participants reporting symptoms or effects by illness phase (includes all questions). All participants from clinic recruitment mode regardless of the number of forms completed.
(PDF)

S3 Fig Heatmaps: Proportion of participants reporting symptoms or effects by illness phase (includes all questions). All participants from community recruitment mode regardless of the number of forms completed.
(PDF)

S4 Fig Heatmaps: Proportion of participants reporting symptoms or effects by illness phase (includes all questions). All participants from contact-cluster recruitment mode regardless of the number of forms completed.
(PDF)

S5 Fig Heatmaps: Proportion of participants reporting symptoms or effects by illness phase (includes all questions). Participants from all recruitment modes from subset of individuals who completed a survey in each illness phase.
(PDF)

S6 Fig HRQoL score by illness phase. (PDF)
