# Supplementary material for: Measuring health related quality of life for dengue patients in Iquitos, Peru
Source: PLoS Negl Trop Dis. 2020 Jul 28;14(7):e0008477. doi: 10.1371/journal.pntd.0008477 (PMC7413550; doi:10.1371/journal.pntd.0008477)
Supplement: S1 Text — (PDF) [file pntd.0008477.s001.pdf]

S1 Text

S1 Text. Original QWB-SA survey in Spanish and English.

Clinic:	Participant ID:	Nickname:	Outcome Visit:	Month:	Day:	Year:

Quality of Well-Being Scale, Self-Administered (QWB-SA), V1.04

Esta encuesta se refiere a problemas de salud que usted haya experimentado en los últimos 3 días, no incluyendo el día de hoy. Por favor responda a todas las preguntas llenando el círculo apropiado con tinta azul o negra. Por favor no use marcas de chequear o bolígrafos de felpa. Gracias.

1. Por favor indique si actualmente tiene algunos de los siguientes síntomas o problemas de salud:

**¿Tiene Ud…**	**SI**	**NO**
a. Pérdida completa de la vista o problemas severos en ambos ojos?	O	O
Pérdida completa de la vista o problemas severos en un solo ojo?	O	O
b. Dificultad al hablar, tal como tartamudear, o no poder hablar	O	O
c. Falta o parálisis de las manos, pies, brazos, o piernas?	O	O
Falta o parálisis de los dedos de las manos o pies?	O	O
d. Alguna deformidad de la cara, dedos, mano o brazo, pie o pierna, o espalda (por ejemplo, escoliosis severo)?	O	O
e. Cansancio general, fatiga o debilidad?	O	O
f. Problema con aumento o pérdida de peso sin desearlo?	O	O
g. Problema con ser de bajo peso o de peso excesivo?	O	O
h. Problemas al masticar su comida adecuadamente?	O	O
i. Pérdida del oído o sordera?	O	O
j. Algún problema de la piel, tal como acné severo, o quemaduras grandes o cicatrices en la cara, cuerpo, brazos, o piernas?	O	O
k. Eczema, salpullido que causa ardor o comezón?	O	O
**¿Cuáles de los siguientes aparatos de salud usa o tiene Ud?**	**SI**	**NO**
a. Dentadura postiza?	O	O
b. Tanque de oxigeno?	O	O
c. Prótesis?	O	O
d. Anteojos o lentes de contacto?	O	O
e. Audífonos?	O	O
f. Lupa?	O	O
g. Soporte para el cuello, espalda o pierna?	O	O

2. En la siguiente lista de problemas, indique en cuales de los últimos 3 días, excluyendo el día de hoy, tuvo el problema. Si no ha tenido el síntoma en los últimos tres días, no deje la pregunta sin contestar, por favor marque la respuesta "Ningún día". Si ha tenido el síntoma en los últimos tres días, por favor marque esos días en que tuvo el problema; si lo tuvo por más de uno de los días, por favor marque todos los días que corresponda.

	**Ningún día**	**Ayer**	**Hace 2 días**	**Hace 3 días**
**Por ejemplo, si tuvo un dolor de cabeza ayer y anteayer, entonces marque lo siguiente: dolor de cabeza?**	O	●	●	O
**¿Tuvo Ud...(Por favor indique todos los días que apliquen)**<br>a. Algún problema con su vista que no se corrije con anteojos o lentes de contacto (tal como doble visión, visión distorsionada, destello (relumbrón) o flotantes)?	O	O	O	O
b. Algún dolor en los ojos, irritación, flujo o sensibilidad excesiva a la luz?	O	O	O	O

© Copyright 1996. Derechos reservados. La modificación, duplicación, o distribución adicional en cualquier forma está estrictamente prohibida sin permiso escrito, Robert M. Kaplan, Theodore G. Ganiats y William J. Sieber. Las preguntas 9a y 9b están disponibles al público a través de derechos de autor en manos de La Corporación RAND.

Clinic:	Participant ID:	Nickname:	Outcome Visit:	Month:	Day:	Year:

¿Tuvo Ud …<br>(Por favor indique todos los días que apliquen)	Ningún día	Ayer	Hace 2 días	Hace 3 días
c. Dolor de cabeza?	O	O	O	O
d. Mareo, dolor o zumbido de oídos?	O	O	O	O
e. Dificultad para oír, flujo o sangrar de un oído?	O	O	O	O
f. Nariz tapada o que fluye o estar sangrando de la nariz?	O	O	O	O
g. Dolor de garganta, dificultad al tragar, o voz ronca?	O	O	O	O
h. Dolor de diente o de la quijada?	O	O	O	O
i. Dolor o estar sangrado de los labios, lengua, o las encías?	O	O	O	O
j. Tos o respiración asmática?	O	O	O	O
k. Respiración corta o dificultad al respirar?	O	O	O	O
l. Dolor de pecho, tensión, palpitaciones, latidos irregulares del corazón, o otra molestia en el pecho?	O	O	O	O
m. Estómago indispuesto, dolor abdominal, nausea, acidez, o vomitar?	O	O	O	O
n. Dificultad al defecar, diarrea, estreñimiento, sangre en el recto, excremento de color negro, o cualquier dolor o molestia en el área del recto?	O	O	O	O
o. Dolor, ardor, o sangre en la orina?	O	O	O	O
p. Pérdida del control de la vejiga, orinar frecuentemente por la noche, o tiene dificultad al orinar?	O	O	O	O
q. Dolor en los órganos sexuales, comezón, ardor o flujo anormal o calambre en el área pélvica o sangrado anormal?	O	O	O	O
r. Mano rota, muñeca, pie, pierna u otro hueso roto (que no sea en la espalda)?	O	O	O	O
s. Dolor, rigidez, calambre, debilidad o adormecimiento en el cuello o espalda?	O	O	O	O
t. Dolor, rigidez, calambre, debilidad o adormecimiento en las caderas o costados?	O	O	O	O
u. Dolor, rigidez, calambre, debilidad o adormecimiento en cualquier coyuntura o músculos de las manos, pies, brazos o piernas?	O	O	O	O
v. Hinchazón de los tobillos, manos, pies o abdomen?	O	O	O	O
w. Fiebre, escalofríos o sudores?	O	O	O	O
x. Pérdida del conocimiento, desmayos, o ataques?	O	O	O	O
y. Dificultad manteniendo su equilibrio, al pararse o caminar?	O	O	O	O

© Copyright 1996. Derechos reservados. La modificación, duplicación, o distribución adicional en cualquier forma está estrictamente prohibida sin permiso escrito, Robert M. Kaplan, Theodore G. Ganiats y William J. Sieber. Las preguntas 9a y 9b están disponibles al público a través de derechos de autor en manos de La Corporación RAND.

Clinic:	Participant ID:	Nickname:	Outcome Visit:	Month:	Day:	Year:

**¿Tuvo Ud...<br>(Por favor indique todos los días que apliquen)**	**Ningún día**	**Ayer**	**Hace 2 días**	**Hace 3 días**
**3. Los siguientes síntomas están relacionados con sus sentimientos, pensamientos y comportamiento. Por favor indique en cuáles de los últimos tres días, excluyendo el día de hoy, ha tenido Ud...**				
a. Dificultad para dormirse o mantener el sueño?	O	O	O	O
b. Momentos de sentirse nervioso o tembloroso?	O	O	O	O
c. Momentos de sentirse trastornado, desanimado, o triste?	O	O	O	O
d. Preocupación o ansiedad excesiva?	O	O	O	O
e. Sentir que tiene poco o no control de los eventos en su vida?	O	O	O	O
f. Sentirse solo o aislado?	O	O	O	O
g. Sentirse frustrado, irritado o enfadado?	O	O	O	O
h. Una cruda o resaca (después de tomar)?	O	O	O	O
i. Interés disminuido o no pudo llevar a cabo el acto sexual?	O	O	O	O
j. Dificultad en entender la palabra escrita o hablada, o pérdida significante de la memoria?	O	O	O	O
k. Pensamientos o imágenes que no pudo sacar de su mente?	O	O	O	O
l. Que tomar algún medicamento incluyendo medicinas no recetadas (aspirina/tylenol, medicinas para alergias, insulina, hormonas, estrógeno, tiroides y prednisone)?	O	O	O	O
m. Que seguir una dieta recetada por un medico por razones de salud?	O	O	O	O
n. Pérdida de apetito o de comer excesivamente?	O	O	O	O
4. En los últimos 3 días, ¿tuvo Ud. algún síntoma, queja de salud, o dolor que no haya mencionado?<br>O Sí O No<br>Si su respuesta es sí ¿cuáles fueron los síntomas y en cuáles días estuvieron presentes?				
Síntoma A:________________________________	O	O	O	O
Síntoma B:________________________________	O	O	O	O

**En los últimos 3 días... (Por favor indique todos los días que apliquen)**	**Ningún día**	**Ayer**	**Hace 2 días**	**Hace 3 días**
5a. ¿Pasó Ud. parte del día o noche como paciente en un hospital, casa de ancianos o centro de rehabilitación?	O	O	O	O
5b. ¿Necesitó ayuda con su cuidado personal, tal como, comer, vestirse, bañarse y caminar dentro de su casa por causa de algún daño o problema de salud?	O	O	O	O
6a. ¿Cuáles de los días manejó un vehículo?	O	O	O	O
6b. ¿Cuáles de los días usó transporte público, tal como un autobús, el metro (tren subterráneo), camioneta médica, tren, o avión?	O	O	O	O
6c. ¿Cuáles de los días no manejó, o no usó transporte público, o necesitó ayuda de otra persona para usarlos por razones relacionadas con su salud?	O	O	O	O

© Copyright 1996. Derechos reservados. La modificación, duplicación, o distribución adicional en cualquier forma está estrictamente prohibida sin permiso escrito, Robert M. Kaplan, Theodore G. Ganiats y William J. Sieber. Las preguntas 9a y 9b están disponibles al público a través de derechos de autor en manos de La Corporación RAND.

Clinic:	Participant ID:	Nickname:	Outcome Visit:	Month:	Day:	Year:

**En los últimos 3 días ¿tuvo Ud... (Por favor indique todos los días que apliquen)?**	**Ningún día**	**Ayer**	**Hace 2 días**	**Hace 3 días**
7a. Dificultad al subir escaleras, usar rampas o caminar fuera de la banqueta?	O	O	O	O
7b. Que evitar caminar, tuvo problemas para caminar, o caminar más despacio que otras personas de su edad?	O	O	O	O
7c. Que cojear o usar un bastón, muletas o soportes metálicos para caminar?	O	O	O	O
7d. Que evitar o tuvo problemas al inclinarse, agacharse o arrodillarse?	O	O	O	O
7e. Problemas al levantar o cargar objetos como libros, maletín, o la compra del supermercado?	O	O	O	O
7f. Alguna otra limitación en sus movimientos físicos?	O	O	O	O
7g. Que pasar toda o la mayor parte del día acostado en una cama, sentado en una silla o sofá por razones de su salud?	O	O	O	O
7h. Que pasar toda o la mayor parte del día en una silla de ruedas?	O	O	O	O
**Si estuvo en una silla de ruedas,** indique en cuales de los días tuvo otra persona que controlar el movimiento de la silla?	O	O	O	O
8a. Por razones de salud physica o emocional, ¿en cuáles de los días evitó, necesitó ayuda, o se sintió limitado en hacer algunas de sus actividades diarias, tal como ir al trabajo, a la escuela o hacer sus quehaceres domésticos?	O	O	O	O
8b. Por razones de salud physica o emocional, ¿en cuáles de los días evitó o se sintió limitado en hacer algunas de sus actividades diarias, tal como visitar a su familia o amigos, su pasatiempo, ir de compras, actividades recreativas o religiosas?	O	O	O	O
8c. En cuáles de los días tuvo que cambiar sus planes o actividades por razones de salud? (Considere solo actividades que no ha mencionado en las últimas 2 preguntas.) ______________________	O	O	O	O

9a. Diría Ud. que su estado de salud es:
- O Excelente
- O Muy Buena
- O Buena
- O Regular
- O Mala

9b. En general, ¿cómo considera Ud. que se encuentra de salud en estos momentos comparado con el año pasado?
- O Mejor que el año pasado
- O Algo mejor que el año pasado
- O Igual que el año pasado
- O Algo peor que el año pasado
- O Mucho peor que el año pasado

9c. Piense en una escala de 0 a 100, cero siendo el estado de salud menos deseado que uno pueda imaginarse y 100 un estado de salud perfecto. En general, ¿qué número de 0 a 100 marcaría usted como su estado de salud en los últimos 3 días?

0	10	20	30	40	50	60	70	80	90	100
O	O	O	O	O	O	O	O	O	O	O

© Copyright 1996. Derechos reservados. La modificación, duplicación, o distribución adicional en cualquier forma está estrictamente prohibida sin permiso escrito, Robert M. Kaplan, Theodore G. Ganiats y William J. Sieber. Las preguntas 9a y 9b están disponibles al público a través de derechos de autor en manos de La Corporación RAND.

Clinic:	Participant ID:	Nickname:	Outcome Visit:	Month:	Day:	Year:

10a.Sexo:

O Masculino
O Femenino

10b Edad:

10c. Cual es su etnicidad (su origen)?

O Africano-Americano
O Asiático o de las Islas Pacíficas
O Anglo-sajón
O Hispano
O Indio Nativo de América
O Otro; por favor indique _______

10d. Cual de los siguientes mejor describe los años de estudio que Ud. ha completado?

O Secundaria
O Preparatoria
O Algo de colegio universitario
O Título universitario (B.S. o B.A)
O Algo de escuela para graduados
O Título Posgraduado (M.A., M.D., PhD.)

© Copyright 1996. Derechos reservados. La modificación, duplicación, o distribución adicional en cualquier forma está estrictamente prohibida sin permiso escrito, Robert M. Kaplan, Theodore G. Ganiats y William J. Sieber. Las preguntas 9a y 9b están disponibles al público a través de derechos de autor en manos de La Corporación RAND.

Clinic:	Participant ID:	Nickname:	Outcome Visit:	Month:	Day:	Year:

Quality of Well-Being Scale, Self-Administered (QWB-SA), V1.04

This survey asks about health problems that you have experienced in the last 3 days, not including today. Please answer all questions by filling in the appropriate circle completely with blue or black ink. Please do not use check marks or felt tip pens. Thank you.

1. Please indicate whether you currently experience each of the following health symptoms or problems:

**Do you have…**	**YES**	**NO**
a. blindness or severely impaired vision in both eyes?	O	O
blindness or severely impaired vision in only one eye?	O	O
b. speech problems such as stuttering, or being unable to speak clearly?	O	O
c. missing or paralyzed hands, feet, arms, or legs?	O	O
missing or paralyzed fingers or toes?	O	O
d. any deformity of the face, fingers, hand or arm, foot or leg, or back (e.g. severe scoliosis)?	O	O
e. general fatigue, tiredness, or weakness?	O	O
f. a problem with unwanted weight gain or weight loss?	O	O
g. a problem with being under or over weight?	O	O
h. problems chewing your food adequately?	O	O
i. any hearing loss or deafness?	O	O
j. any noticeable skin problems, such as bad acne or large burns or scars on face, body, arms, or legs?	O	O
k. eczema or burning/itching rash?	O	O

**Which of the following health aides do you use/have?**	**YES**	**NO**
a. dentures?	O	O
b. oxygen tank?	O	O
c. prosthesis?	O	O
d. eye glasses or contact lenses?	O	O
e. hearing aide?	O	O
f. magnifying glass?	O	O
g. neck, back, or leg brace?	O	O

2. For the following list of problems, indicate which days (if any) over the past 3 days, not including today, you had the problem. If you have not had the symptom in the past 3 days, do not leave the question blank, please fill in "no days." If you have experienced the symptom in the past 3 days, please fill in which of the days you had it; if you experienced it on more than one of the days, please fill in all days that apply.

	**No days**	**Yester–day**	**2 days ago**	**3 days ago**
**For example, if you had a headache yesterday and the day before that, you would mark for a headache:**	O	●	●	O
**Did you have…(please fill in all days that apply)**<br>a. any problems with your vision not corrected with glasses or contact lenses (such as double vision, distorted vision, flashes, or floaters)?	O	O	O	O
b. any eye pain, irritation, discharge, or excessive sensitivity to light?	O	O	O	O

© Copyright 1996, All Rights Reserved. Modification, duplication, or further distribution in any form strictly prohibited without written permission, Robert M. Kaplan, Theodore G. Ganiats, and William J. Sieber. Questions 9a and 9b are publicly available through copyright held by THE RAND CORP.

Clinic:	Participant ID:	Nickname:	Outcome Visit:	Month:	Day:	Year:

Did you have… (please fill in all days that apply)	No days	Yester–day	2 days ago	3 days ago
c. a headache?	O	O	O	O
d. dizziness, earache, or ringing in your ears?	O	O	O	O
e. difficulty hearing, or discharge, or bleeding from an ear?	O	O	O	O
f. stuffy or runny nose, or bleeding from the nose?	O	O	O	O
g. a sore throat, difficulty swallowing, or hoarse voice?	O	O	O	O
h. a tooth ache or jaw pain?	O	O	O	O
i. sore or bleeding lips, tongue, or gums?	O	O	O	O
j. coughing or wheezing?	O	O	O	O
k. shortness of breath or difficulty breathing?	O	O	O	O
l. chest pain, pressure, palpitations, fast or skipped heart beat, or other discomfort in the chest?	O	O	O	O
m. an upset stomach, abdominal pain, nausea, heartburn, or vomiting?	O	O	O	O
n. difficulty with bowel movements, diarrhea, constipation, rectal bleeding, black tar-like stools, or any pain or discomfort in the rectal area?	O	O	O	O
o. pain, burning, or blood in urine?	O	O	O	O
p. loss of bladder control, frequent night-time urination, or difficulty with urination?	O	O	O	O
q. genital pain, itching, burning, or abnormal discharge, or pelvic cramping or abnormal bleeding (does not include normal menstruation)?	O	O	O	O
r. a broken arm, wrist, foot, leg, or any other broken bone (other than in the back)?	O	O	O	O
s. pain, stiffness, cramps, weakness, or numbness *in the neck or back*?	O	O	O	O
t. pain, stiffness, cramps, weakness, or numbness *in the hips or sides*?	O	O	O	O
u. pain, stiffness, cramps, weakness, or numbness in any *of the joints or muscles of the hand, feet, arms, or legs*?	O	O	O	O
v. swelling of ankles, hands, feet, or abdomen?	O	O	O	O
w. fever, chills, or sweats?	O	O	O	O
x. loss of consciousness, fainting, or seizures?	O	O	O	O
y. difficulty with your balance, standing, or walking?	O	O	O	O

© Copyright 1996, All Rights Reserved. Modification, duplication, or further distribution in any form strictly prohibited without written permission, Robert M. Kaplan, Theodore G. Ganiats, and William J. Sieber. Questions 9a and 9b are publicly available through copyright held by THE RAND CORP.

Clinic:	Participant ID:	Nickname:	Outcome Visit:	Month:	Day:	Year:

**Did you have… (please fill in all days that apply)**	**No days**	**Yester-day**	**2 days ago**	**3 days ago**
**3. The following symptoms are about your feelings, thoughts, and behaviors. Please fill in which days (if any) over the past 3 days, not including today, you have had…**				
a. trouble falling asleep or staying asleep?	O	O	O	O
b. spells of feeling nervous or shaky?	O	O	O	O
c. spells of feeling upset, downhearted, or blue?	O	O	O	O
d. excessive worry or anxiety?	O	O	O	O
e. feelings that you had little or no control over events in your life?	O	O	O	O
f. feelings of being lonely or isolated?	O	O	O	O
g. feelings of frustration, irritation, or close to losing your temper?	O	O	O	O
h. a hangover?	O	O	O	O
i. any decrease of sexual interest or performance?	O	O	O	O
j. confusion, difficulty understanding the written or spoken word, or significant memory loss?	O	O	O	O
k. thoughts or images you could not get out of your mind?	O	O	O	O
l. to take any medication including over-the-counter remedies (aspirin/tylenol, allergy medications, insulin, hormones, estrogen, thyroid, prednisone)?	O	O	O	O
m. to stay on a medically prescribed diet for health reasons?	O	O	O	O
n. a loss of appetite or over-eating?	O	O	O	O

4. In the last 3 days did you have any symptoms, health complaints, or pains that have not been mentioned?

O Yes O No

If yes, what were they and on which days did you have them?

	No days	Yester-day	2 days ago	3 days ago
Symptom A:________________________________	O	O	O	O
Symptom B:________________________________	O	O	O	O

**Over the last 3 days…(please fill in all days that apply)**	**No days**	**Yester-day**	**2 days ago**	**3 days ago**
5a. did you spend any part of the day or night as a patient in a hospital, nursing home, or rehabilitation center?	O	O	O	O
5b. because of any impairment or health problem, did you need help with your personal care needs, such as eating, dressing, bathing, or getting around your home?	O	O	O	O
6a. which days did you drive a motor vehicle?	O	O	O	O
6b. which days did you use public transportation such as a bus, subway, Medi-van, train, or airplane?	O	O	O	O
6c. which days did you either not drive a motor vehicle or not use public transportation because of your health, or need help from another person to use?	O	O	O	O

© Copyright 1996, All Rights Reserved. Modification, duplication, or further distribution in any form strictly prohibited without written permission, Robert M. Kaplan, Theodore G. Ganiats, and William J. Sieber. Questions 9a and 9b are publicly available through copyright held by THE RAND CORP.

Clinic:	Participant ID:	Nickname:	Outcome Visit:	Month:	Day:	Year:

Over the last three days, did you… (please fill in all days that apply)	No days	Yester-day	2 days ago	3 days ago
7a. have trouble climbing stairs or inclines or walking off the curb?	O	O	O	O
7b. avoid walking, have trouble walking, or walk more slowly than other people your age?	O	O	O	O
7c. limp or use a cane, crutches, or walker?	O	O	O	O
7d. avoid or have trouble bending over, stooping, or kneeling?	O	O	O	O
7e. have any trouble lifting or carrying everyday objects such as books, a briefcase, or groceries?	O	O	O	O
7f. have any other limitations in physical movements?	O	O	O	O
7g. spend all or most of the day in a bed, chair, or couch because of health reasons?	O	O	O	O
7h. spend all or most of the day in a wheelchair?	O	O	O	O
**If in a wheelchair**, on which days did someone else control its movement?	O	O	O	O
8a. because of any physical or emotional health reasons, on which days did you avoid, need help with, or were limited in doing some of your usual activities, such as work, school, or housekeeping?	O	O	O	O
8b. because of physical or emotional health reasons, on which days did you avoid or feel limited in doing some of your usual activities, such as visiting family or friends, hobbies, shopping, recreational, or religious activities?	O	O	O	O
8c. on which days did you have to change any of your plans or activities because of your health? (Consider only activities that you did not report in the last 2 questions.)	O	O	O	O

9a. Would you say that your health is:
- O Excellent
- O Very Good
- O Good
- O Fair
- O Poor

9b. Compared to a year ago, how would you rate your health in general now?
- O Much better now than a year ago
- O Somewhat better now than one year ago
- O About the same as a year ago
- O Somewhat worse than a year ago
- O Much worse than a year ago

9c. Think about a scale of 0 to 100, with zero being the least desirable state of health that you could imagine and 100 being perfect health. What number from 0 to 100 would you give to the state of your health, on average, over the last 3 days?

0	10	20	30	40	50	60	70	80	90	100
O	O	O	O	O	O	O	O	O	O	O

10a. Sex: O Male
O Female

10b. Age in Years: [ ][ ][ ]

10c. What is your ethnicity?
- O African American
- O Asian/Pacific Islander
- O Caucasian – Non Hispanic
- O Hispanic
- O Native American
- O Other

10d. Which of the following best describes your educational background?
- O 8th Grade Graduate
- O High School Graduate
- O Some College
- O College Graduate (B.S. or B.A. degree)
- O Some Graduate School
- O Completed Post-Graduate (M.A.,M.D.,Ph.D.)

© Copyright 1996, All Rights Reserved. Modification, duplication, or further distribution in any form strictly prohibited without written permission, Robert M. Kaplan, Theodore G. Ganiats, and William J. Sieber. Questions 9a and 9b are publicly available through copyright held by THE RAND CORP.
