# Supplementary material for: Measuring health related quality of life for dengue patients in Iquitos, Peru
Source: PLoS Negl Trop Dis. 2020 Jul 28;14(7):e0008477. doi: 10.1371/journal.pntd.0008477 (PMC7413550; doi:10.1371/journal.pntd.0008477)
Supplement: S1 Table — (PDF) [file pntd.0008477.s002.pdf]

S1 Table.

Survey questions and question groupings and original survey in Spanish and English

- **Columns**
  - **Group:** Groupings of questions for results presentation
  - **English:** The official English phrasing of the question in QWB-SA
  - **Spanish:** The official Spanish phrasing of the question in QWB-SA
  - **Abbreviated:** for used in the figures
- **Notes***
  - The question asking about the presence of a hangover (directly as a consequence of alcohol) was misinterpreted by our field teams as being dehydrated or feeling thirsty. This was detected at too late a stage to correct, but is likely to have had a minimal impact on HRQoL scores given the quantity of questions in the survey.
  - Question numbers are demarcated by light grey horizontal bars

Group	English	Spanish	Abbreviated
**QUESTION 1**			
Physical	blindness or severely impaired vision in both eyes?	Pérdida completa de la vista o problemas severos en ambos ojos?	Blindness (bilateral)
Physical	blindness or severely impaired vision in only one eye?	Pérdida completa de la vista o problemas severos en un solo ojo?	Blindness (unilateral)
Physical	speech problems such as stuttering, or being unable to speak clearly?	Dificultad al hablar, tal como tartamudear, o no poder hablar	Speech problem
Physical	missing or paralyzed hands, feet, arms, or legs?	Falta o parálisis de las manos, pies, brazos, o piernas?	Paralysis of limb
Physical	missing or paralyzed fingers or toes?	Falta o parálisis de los dedos de las manos o pies?	Paralysis of digit
Physical	any deformity of the face, fingers, hand or arm, foot or leg, or back (e.g. severe scoliosis)?	Alguna deformidad de la cara, dedos, mano o brazo, pie o pierna, o espalda (por ejemplo, escoliosis severo)?	Deformity
Physical	general fatigue, tiredness, or weakness?	Cansancio general, fatiga o debilidad?	Fatigue
Physical	a problem with unwanted weight gain or weight loss?	Problema con aumento o pérdida de peso sin desearlo?	Weight change
Physical	a problem with being under or over weight?	Problema con ser de bajo peso o de peso excesivo?	Over or under weight
Physical	problems chewing your food adequately?	Problemas al masticar su comida adecuadamente?	Chewing problem
Physical	any hearing loss or deafness?	Pérdida del oído o sordera?	Deafness
Physical	any noticeable skin problems, such as bad acne or large burns or scars on face, body, arms, or legs?	Algún problema de la piel, tal como acné severo, o quemaduras grandes o cicatrices en la cara, cuerpo, brazos, o piernas?	Skin condition
Physical	eczema or burning/itching rash?	Eczema, salpullido que causa ardor o comezón?	Itching
Physical	**Have used the following**		
Physical	dentures?	Dentadurapostiza?	

Physical	oxygen tank?	Tanquede oxigeno?	
Physical	prosthesis?	Prótesis?	
Physical	eye glasses or contact lenses?	Anteojoso lentes de contacto?	
Physical	hearing aide?	Audífonos?	
Physical	magnifying glass?	Lupa?	
Physical	neck, back, or leg brace?	Soporte para el cuello, espalda o pierna?	
**QUESTION 2**			
Physical	a. any problems with your vision not corrected with glasses or contact lenses (such as double vision, distorted vision, flashes, or floaters)?	a. Algún problema con su vista que no se corrije con anteojos o lentes de contacto (tal como doble visión, visión distorsionada, destello (relumbrón) o flotantes)?	Visual problem
Physical	any eye pain, irritation, discharge, or excessive sensitivity to light?	Algún dolor en los ojos, irritación, flujo o sensibilidad excesiva ala luz?	Eye pain
Physical	a headache?	Dolor de cabeza?	Headache
Physical	dizziness, earache, or ringing in your ears?	Mareo, dolor o zumbido de oídos?	Dizziness
Physical	difficulty hearing, or discharge, or bleeding from an ear?	Dificultad para oír, flujo o sangrar de un oído?	Ear symptoms
Physical	stuffy or runny nose, or bleeding from the nose?	Nariz tapada o que fluye o estar sangrando de la nariz?	Nose symptoms
Physical	a sore throat, difficulty swallowing, or hoarse voice?	Dolor de garganta, dificultad al tragar, o voz ronca?	Sore throat
Physical	a tooth ache or jaw pain?	Dolor de diente o de la quijada?	Toothache
Physical	sore or bleeding lips, tongue, or gums?	Dolor o estar sangrado de los labios, lengua, o las encías?	Oral bleed
Physical	coughing or wheezing?	Tos o respiración asmática?	Cough
Physical	shortness of breath or difficulty breathing?	Respiración corta o dificultad al respirar?	Shortness of breath
Physical	chest pain, pressure, palpitations, fast or skipped heartbeat, or other discomfort in the chest?	Dolorde pecho, tensión, palpitaciones, latidos irregulares del corazón, o otra molestia en el pecho?	Chest pain
Physical	an upset stomach, abdominal pain, nausea, heartburn, or vomiting?	Estómago indispuesto, dolor abdominal, nausea, acidez,o vomitar?	Abdominal pain
Physical	difficulty with bowel movements, diarrhea, constipation, rectal bleeding, black tar-like stools, or any pain or discomfort in the rectal area?	Dificultad al defecar, diarrea, estreñimiento, sangre en el recto, excremento de color negro, o cualquier dolor o molestia en el áreadel recto?	Change bowels
Physical	pain, burning, or blood in urine?	Dolor, ardor, o sangre en la orina?	Dysuria
Physical	loss of bladder control, frequent night-time urination, or difficulty with urination?	Pérdida del control de la vejiga, orinar frecuentemente por la noche, o tiene dificultad al orinar?	Reduced bladder control
Physical	genital pain, itching, burning, or abnormal discharge, or pelvic cramping or abnormal bleeding (does not include normal menstruation)?	Dolor en los órganos sexuales, comezón, ardor o flujo anormal o calambre en el área pélvica o sangrado anormal?	Genital symptoms

Physical	a broken arm, wrist, foot, leg, or any other broken bone (other than in the back)?	Mano rota, muñeca, pie, pierna u otro hueso roto (que no sea en la espalda)?	Broken bones
Physical	pain, stiffness, cramps, weakness, or numbness in the neck or back?	Dolor, rigidez, calambre, debilidad o adormecimiento en el cuello o espalda?	Back or neck pain
Physical	pain, stiffness, cramps, weakness, or numbness in the hips or sides?	Dolor, rigidez, calambre, debilidad o adormecimiento en las caderas o costados?	Hip pain
Physical	pain, stiffness, cramps, weakness, or numbness in any of the joints or muscles of the hand, feet, arms, or legs?	Dolor, rigidez, calambre, debilidad o adormecimiento en cualquier coyuntura o músculos de las manos, pies, brazos o piernas?	Joint pain
Physical	swelling of ankles, hands, feet, or abdomen?	Hinchazón de los tobillos, manos, pies o abdomen?	Edema
Physical	fever, chills, or sweats?	Fiebre, escalofríos o sudores?	Fever or chills
Physical	loss of consciousness, fainting, or seizures?	Pérdida del conocimiento, desmayos, o ataques?	Loss of consciousness
Physical	difficulty with your balance, standing, or walking?	Dificultad manteniendo su equilibrio, al pararse o caminar?	Balance problem
**QUESTION 3**			
Psychological	a hangover?	Una cruda o resaca (despuésde tomar)?	Hungover *
Psychological	any decrease of sexual interest or performance?	Interés disminuido o no pudo llevar a cabo el acto sexual?	Reduced libido
Psychological	confusion, difficulty understanding the written or spoken word, or significant memory loss?	Dificultad en entender la palabra escrita o hablada, o pérdida significante de la memoria?	Confusion
Psychological	thoughts or images you could not get out of your mind?	Pensamientos o imágenes que no pudo sacar de su mente?	Invasive thoughts
Psychological	feelings of being lonely or isolated?	Sentirse solo o aislado?	Loneliness
Psychological	feelings of frustration, irritation, or close to losing your temper?	Sentirse frustrado, irritado o enfadado?	Frustration
Psychological	trouble falling asleep or staying asleep?	Dificultad para dormirse o mantener el sueño?	Insomnia
Psychological	spells of feeling nervous or shaky?	Momentos de sentirse nervioso o tembloroso?	Nervousness
Psychological	spells of feeling upset, downhearted, or blue?	Momentos de sentirse trastornado, desanimado, o triste?	Feeling upset
Psychological	excessive worry or anxiety?	Preocupació no ansiedad excesiva?	Anxiety
Psychological	feelings that you had little or no control over events in your life?	Sentir que tiene poco o no control de los eventos en su vida?	No control of life
Psychological	a loss of appetite or over-eating?	Pérdida de apetito o de comer excesivamente?	Anorexia
**QUESTION 4**			
	In the last 3 days did you have any symptoms, health complaints, or pains that have not been mentioned?	En los últimos 3 días, ¿tuvo Ud. algún síntoma, queja de salud, o dolor que no haya mencionado?	
**QUESTION 5**			
self-care	did you spend any part of the day or night as a patient in a hospital, nursing home, or rehabilitation center?	¿Pasó Ud. parte del día o noche como paciente en un hospital, casa de ancianos o centro de rehabilitación?	Hospital care
self-care	because of any impairment or health problem, did you need help	¿Necesitó ayuda con su cuidado personal, tal como, comer,	Help with personal care

	with your personal care needs, such as eating, dressing, bathing, or getting around your home?	vestirse, bañarse y caminar dentro de su casa por causa de algún daño o problema de salud?	
**QUESTION 6**			
mobility & physical functioning	which days did you drive a motor vehicle?	¿Cuáles de los días manejó un vehículo?	Drove vehicle
mobility & physical functioning	which days did you use public transportation such as a bus, subway, Medi-van, train, or airplane?	¿Cuáles de los días usó transporte público, tal como un autobús, el metro (tren subterráneo), camioneta médica, tren, o avión?	Used public transport
mobility & physical functioning	which days did you either not drive a motor vehicle or not use public transportation because of your health, or need help from another person to use?	¿Cuáles de los días no manejó, o no usó transporte público, o necesitó ayuda de otra persona para usarlos por razones relacionadas con su salud?	Transport not used
**QUESTION 7**			
mobility & physical functioning	have trouble climbing stairs or inclines or walking off the curb?	Dificultad al subir escaleras, usar rampas o caminar fuera de la banqueta?	Difficulty with stairs
mobility & physical functioning	Avoid walking, have trouble walking, or walk more slowly than other people your age?	Que evitar caminar, tuvo problemas para caminar, o caminar más despacio que otras personas de su edad?	Avoid walking
mobility & physical functioning	limp or use a cane, crutches, or walker?	Que cojear o usar un bastón, muletas o soportes metálicos para caminar?	Limp or walking aid
mobility & physical functioning	avoid or have trouble bending over, stooping, or kneeling?	Que evitar o tuvo problemas al inclinarse, agacharse o arrodillarse?	Difficulty bending
mobility & physical functioning	have any trouble lifting or carrying everyday objects such as books, a briefcase, or groceries?	Problemas al levantar o cargar objetos como libros, maletín, o la compra del supermercado?	Difficulty carrying
mobility & physical functioning	have any other limitations in physical movements?	Alguna otra limitación en sus movimientos físicos?	Other physical
mobility & physical functioning	spend all or most of the day in a bed, chair, or couch because of health reasons?	Que pasar toda o la mayor parte del día acostado en una cama, sentado en una silla o sofá por razones de su salud?	Bedbound
mobility & physical functioning	spend all or most of the day in a wheelchair?	Que pasar toda o la mayor parte del día en una silla de ruedas?	Used wheelchair
mobility & physical functioning	If in a wheelchair, on which days did someone else control its movement?	Si estuvo en una silla de ruedas, indique en cuales de los días tuvo otra persona que controlar el movimiento de la silla?	Wheelchair controlled other
**QUESTION 8**			

usual social activity	because of any physical or emotional health reasons, on which days did you avoid, need help with, or were limited in doing some of your usual activities, such as work, school, or housekeeping?	Por razones de salud physica o emocional, ¿en cuáles de los días evitó, necesitó ayuda, o se sintió limitado en hacer algunas de sus actividades diarias, tal como ir al trabajo, a la escuela o hacer sus quehaceres domésticos?	Affect school/work
usual social activity	because of physical or emotional health reasons, on which days did you avoid or feel limited in doing some of your usual activities, such as visiting family or friends, hobbies, shopping, recreational, or religious activities?	Por razones de salud physica o emocional, ¿en cuáles de los días evitó o se sintió limitado en hacer algunas de sus actividades diarias, tal como visitar a su familia o amigos, su pasatiempo, ir de compras, actividades recreativas o religiosas?	Affect personal life
usual social activity	on which days did you have to change any of your plans or activities because of your health? (Consider only activities that you did not report in the last 2 questions.)	En cuáles de los días tuvo que cambiar sus planes o actividades por razones de salud? (Considere solo actividades que no ha mencionado en las últimas 2 preguntas.)	Change plans
