# Supplementary material for: Measuring health related quality of life for dengue patients in Iquitos, Peru
Source: PLoS Negl Trop Dis. 2020 Jul 28;14(7):e0008477. doi: 10.1371/journal.pntd.0008477 (PMC7413550; doi:10.1371/journal.pntd.0008477)
Supplement: S2 Table — (PDF) [file pntd.0008477.s003.pdf]

<u>S2 Table</u>

Participant characteristics for subset of individuals who completed a survey in each study phase.

Characteristic	All	Clinic	Community	Cluster
Number of cases	50	6	31	13
Number of forms	150	18	93	39
Sex (%)				
Male	25 (50%)	1 (17%)	18 (58%)	6 (46%)
Female	25 (50%)	5 (83%)	13 (42%)	7 (54%)
Median age (IQR)	15.5 (12-21.75)	30 (22.25-37)	14 (11.5-17.5)	12 (10-34)
Median symptom duration in days (IQR)*	1.5 (1-3)	1.5 (1-2.75)	2 (1-3)	1 (1-1)
Hospitalized (%)	5 (10%)	1 (17%)	3 (10%)	1 (8%)
Serotype (%)				
DENV2	48 (96%)	5 (83%)	31 (100%)	12 (92%)
DENV3	2 (4%)	1 (17%)	0 (0%)	1 (8%)

*Median number of days of symptoms a participant had already experienced on the day the diagnostic blood sample was taken.
