# Supplementary material for: Measuring health related quality of life for dengue patients in Iquitos, Peru
Source: PLoS Negl Trop Dis. 2020 Jul 28;14(7):e0008477. doi: 10.1371/journal.pntd.0008477 (PMC7413550; doi:10.1371/journal.pntd.0008477)
Supplement: S3 Table — (PDF) [file pntd.0008477.s004.pdf]

S3 Table

Frequency (%) of all physical and psychological symptoms and social limitations by phase of illness.

- Pair-wise comparisons of proportions between early-acute and convalescent and late-acute and convalescent illness phases.
- This includes all participants from all recruitment modes.
- * Fisher's exact test: p<0.05 corrected to 0.0007 with Bonferroni correction,
- ** Fisher's exact test: p<0.01 05 corrected to 0.0001 with Bonferroni correction.

	EARLY-ACUTE (EA)	*LATE-ACUTE (LA)*	*CONVALESCENT (C)*	*P-VALUE*	*P-VALUE*	*P-VALUE*
PHYSICAL	**(n=69)**	**(n=67)**	**(n=59)**	**EA v LA**	**EA v C**	**LA v C**
Fever or chills	58 (84)	14 (21)	1 (2)	0.000**	0.000**	0.001*
Headache	55 (80)	19 (28)	7 (12)	0.000**	0.000**	0.028
Fatigue	52 (75)	28 (42)	5 (8)	0.000*	0.000**	0.000**
Anorexia	36 (52)	14 (21)	1 (2)	0.000*	0.000**	0.001*
Abdominal pain	36 (52)	18 (27)	5 (8)	0.003	0.000**	0.010
Eye pain	30 (43)	12 (18)	2 (3)	0.002	0.000**	0.011
Weight change	28 (41)	11 (16)	2 (3)	0.002	0.000**	0.019
Dizziness	32 (46)	11 (16)	4 (7)	0.000*	0.000**	0.108
Change bowels	19 (28)	7 (10)	0 (0)	0.016	0.000**	0.014
Itching	30 (43)	26 (39)	7 (12)	0.605	0.000**	0.001*
Balance problem	14 (20)	5 (7)	0 (0)	0.046	0.000**	0.060
Sore throat	19 (28)	8 (12)	2 (3)	0.031	0.000*	0.102
Chest pain	15 (22)	8 (12)	1 (2)	0.170	0.001	0.036
Cough	18 (26)	10 (15)	3 (5)	0.138	0.002	0.084
Back or neck pain	9 (13)	10 (15)	0 (0)	0.808	0.004	0.002
Joint pain	9 (13)	6 (9)	1 (2)	0.586	0.020	0.120
Shortness of breath	9 (13)	3 (4)	1 (2)	0.128	0.020	0.622
Toothache	6 (9)	3 (4)	0 (0)	0.493	0.030	0.247
Visual problem	6 (9)	1 (1)	0 (0)	0.116	0.030	1.000
Chewing problem	9 (13)	3 (4)	2 (3)	0.128	0.063	1.000
Took medication	8 (12)	2 (3)	2 (3)	0.097	0.106	1.000
Dysuria	6 (9)	4 (6)	1 (2)	0.745	0.123	0.370
Oral bleeding	4 (6)	3 (4)	0 (0)	1.000	0.124	0.247
Nose symptoms	8 (12)	5 (7)	3 (5)	0.562	0.222	0.722
Hip pain	6 (9)	5 (7)	2 (3)	1.000	0.286	0.447
Ear symptoms	2 (3)	1 (1)	0 (0)	1.000	0.499	1.000
Edema	2 (3)	2 (3)	0 (0)	1.000	0.499	0.498
Blindness (unilateral)	1 (1)	0 (0)	2 (3)	1.000	0.595	0.217
Deformity	1 (1)	0 (0)	2 (3)	1.000	0.595	0.217

Paralysis of digits	2 (3)	2 (3)	3 (5)	1.000	0.661	0.664
Blindness (bilateral)	4 (6)	0 (0)	2 (3)	0.120	0.686	0.217
Over or under weight	4 (6)	2 (3)	2 (3)	0.681	0.686	1.000
Skin condition	4 (6)	1 (1)	2 (3)	0.366	0.686	0.599
Deafness	2 (3)	0 (0)	2 (3)	0.496	1.000	0.217
Genital symptoms	2 (3)	1 (1)	1 (2)	1.000	1.000	1.000
Loss of consciousness	1 (1)	2 (3)	0 (0)	0.617	1.000	0.498
Paralysis of limbs	3 (4)	0 (0)	2 (3)	0.245	1.000	0.217
Reduced bladder control	1 (1)	2 (3)	0 (0)	0.617	1.000	0.498
Speech problem	2 (3)	1 (1)	2 (3)	1.000	1.000	0.599
Broken bones	0 (0)	0 (0)	0 (0)	NA	NA	NA
PSYCHOLOGICAL						
Insomnia	33 (48)	23 (34)	3 (5)	0.120	0.000**	0.000**
Feeling upset	28 (41)	16 (24)	2 (3)	0.045	0.000**	0.002
Nervousness	21 (30)	13 (19)	2 (3)	0.167	0.000**	0.006
*Hungover^*	19 (28)	5 (7)	2 (3)	0.003	0.000*	0.447
No control of life	17 (25)	6 (9)	2 (3)	0.021	0.001	0.281
Frustration	20 (29)	10 (15)	4 (7)	0.063	0.001	0.167
Loneliness	13 (19)	3 (4)	1 (2)	0.015	0.002	0.622
Anxiety	21 (30)	12 (18)	5 (8)	0.110	0.002	0.190
Reduced libido	8 (12)	5 (7)	0 (0)	0.562	0.007	0.060
Invasive thoughts	6 (9)	2 (3)	2 (3)	0.275	0.286	1.000
Confusion	1 (1)	1 (1)	0 (0)	1.000	1.000	1.000
SELF-CARE						
Hospitalized	11 (16)	13 (19)	0 (0)	0.657	0.001	0.000*
Help with personal care	9 (13)	6 (9)	0 (0)	0.586	0.004	0.029
USUAL SOCIAL ACTIVITY						
Affect school/work	35 (51)	14 (21)	0 (0)	0.000*	0.000**	0.000**
Affect personal life	31 (45)	19 (28)	0 (0)	0.052	0.000**	0.000**
Change plans	23 (33)	18 (27)	0 (0)	0.458	0.000**	0.000**
MOBILITY & PHYSICAL FUNCTIONING						
Bedbound	36 (52)	15 (22)	2 (3)	0.000*	0.000**	0.002
Avoid walking	22 (32)	7 (10)	0 (0)	0.003	0.000**	0.014
Difficulty bending	20 (29)	4 (6)	0 (0)	0.001*	0.000**	0.122
Difficulty carrying	18 (26)	6 (9)	2 (3)	0.012	0.000*	0.281
Other physical limitation	10 (14)	2 (3)	0 (0)	0.031	0.002	0.498
Difficulty with stairs	11 (16)	5 (7)	1 (2)	0.183	0.006	0.213
On medical diet	6 (9)	2 (3)	0 (0)	0.275	0.030	0.498
Transport not used	12 (17)	12 (18)	3 (5)	1.000	0.051	0.030

Drove vehicle	4 (6)	6 (9)	10 (17)	0.528	0.051	0.193
Used public transport	32 (46)	30 (45)	24 (41)	0.865	0.593	0.719
Limp or walking aids	1 (1)	0 (0)	0 (0)	1.000	1.000	NA
Used wheelchair	1 (1)	0 (0)	0 (0)	1.000	1.000	NA
Wheelchair controlled by. other	1 (1)	0 (0)	0 (0)	1.000	1.000	NA
