# Supplementary material for: Measuring health related quality of life for dengue patients in Iquitos, Peru
Source: PLoS Negl Trop Dis. 2020 Jul 28;14(7):e0008477. doi: 10.1371/journal.pntd.0008477 (PMC7413550; doi:10.1371/journal.pntd.0008477)
Supplement: S4 Table — (PDF) [file pntd.0008477.s005.pdf]

S4 Table

HRQoL Scores by illness phase and recruitment mode only for subset of individuals who completed a survey in each study phase.

recruitment	Participants	phase	median score (IQR*)	range
All	50	early_acute	0.52 (0.40-0.62)	0.25-0.74
		late_acute	0.75 (0.63-1.00)	0.26-1.00
		convalescent	1.00 (0.80-1.00)	0.48-1.00
Clinic	6	early_acute	0.38 (0.29-0.51)	0.25-0.57
		late_acute	0.69 (0.57-0.94)	0.26-1.00
		convalescent	0.77 (0.74-0.81)	0.62-1.00
Community	31	early_acute	0.54 (0.40-0.62)	0.26-0.74
		late_acute	0.75 (0.65-0.98)	0.36-1.00
		convalescent	1.00 (0.84-1.00)	0.48-1.00
Contact	13	early_acute	0.56 (0.48-0.62)	0.26-0.70
		late_acute	0.77 (0.63-1.00)	0.45-1.00
		convalescent	1.00 (0.89-1.00)	0.48-1.00

*IQR: Interquartile range
