# Supplementary material for: Measuring health related quality of life for dengue patients in Iquitos, Peru
Source: PLoS Negl Trop Dis. 2020 Jul 28;14(7):e0008477. doi: 10.1371/journal.pntd.0008477 (PMC7413550; doi:10.1371/journal.pntd.0008477)
Supplement: S1 Fig — All participants from all recruitment modes regardless of the number of forms completed. (PDF) [file pntd.0008477.s006.pdf]

S1 Figure

Heatmaps: Proportion of participants reporting symptoms or effects by illness phase (includes all questions)

Figure S1: **All participants from all recruitment modes regardless of the number of forms completed** (left column: early-acute, middle column: late-acute, right column: convalescent phase). The proportion is represented by the redness of the cell and the actual proportion is shown by the number in each cell. * Fisher's exact test: *P<0.05*, ** Fisher's exact test: *P<0.01.*

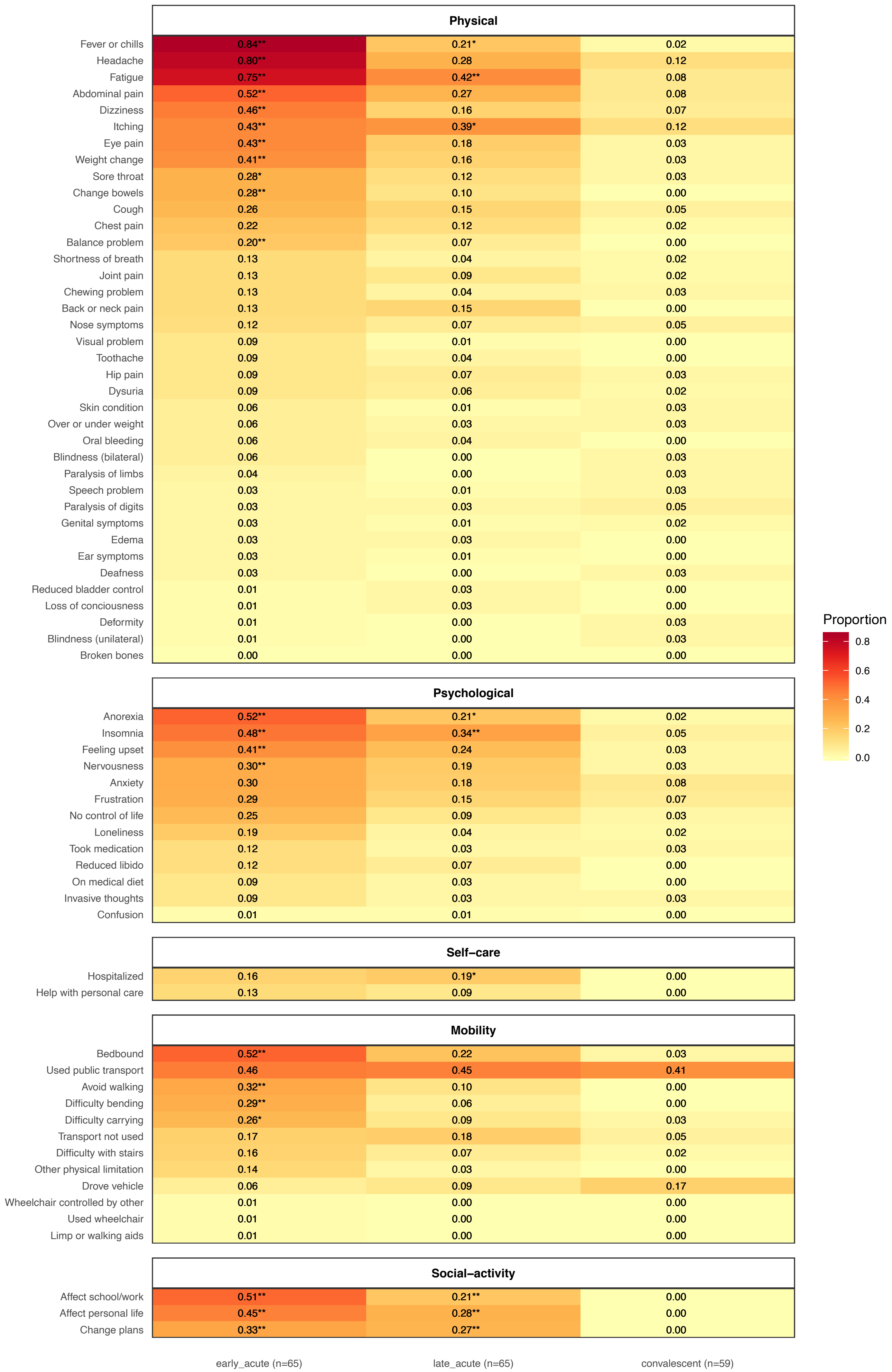
Physical
Fever or chills 0.84** 0.21* 0.02
Headache 0.80** 0.28 0.12
Fatigue 0.75** 0.42** 0.08
Abdominal pain 0.52** 0.27 0.08
Dizziness 0.46** 0.16 0.07
Itching 0.43** 0.39* 0.12
Eye pain 0.43** 0.18 0.03
Weight change 0.41** 0.16 0.03
Sore throat 0.28* 0.12 0.03
Change bowels 0.28** 0.10 0.00
Cough 0.26 0.15 0.05
Chest pain 0.22 0.12 0.02
Balance problem 0.20** 0.07 0.00
Shortness of breath 0.13 0.04 0.02
Joint pain 0.13 0.09 0.02
Chewing problem 0.13 0.04 0.03
Back or neck pain 0.13 0.15 0.00
Nose symptoms 0.12 0.07 0.05
Visual problem 0.09 0.01 0.00
Toothache 0.09 0.04 0.00
Hip pain 0.09 0.07 0.03
Dysuria 0.09 0.06 0.02
Skin condition 0.06 0.01 0.03
Over or under weight 0.06 0.03 0.03
Oral bleeding 0.06 0.04 0.00
Blindness (bilateral) 0.06 0.00 0.03
Paralysis of limbs 0.04 0.00 0.03
Speech problem 0.03 0.01 0.03
Paralysis of digits 0.03 0.03 0.05
Genital symptoms 0.03 0.01 0.02
Edema 0.03 0.03 0.00
Ear symptoms 0.03 0.01 0.00
Deafness 0.03 0.00 0.03
Reduced bladder control 0.01 0.03 0.00
Loss of conciousness 0.01 0.03 0.00
Deformity 0.01 0.00 0.03
Blindness (unilateral) 0.01 0.00 0.03
Broken bones 0.00 0.00 0.00
Psychological
Anorexia 0.52** 0.21* 0.02
Insomnia 0.48** 0.34** 0.05
Feeling upset 0.41** 0.24 0.03
Nervousness 0.30** 0.19 0.03
Anxiety 0.30 0.18 0.08
Frustration 0.29 0.15 0.07
No control of life 0.25 0.09 0.03
Loneliness 0.19 0.04 0.02
Took medication 0.12 0.03 0.03
Reduced libido 0.12 0.07 0.00
On medical diet 0.09 0.03 0.00
Invasive thoughts 0.09 0.03 0.03
Confusion 0.01 0.01 0.00
Self–care
Hospitalized 0.16 0.19* 0.00
Help with personal care 0.13 0.09 0.00
Mobility
Bedbound 0.52** 0.22 0.03
Used public transport 0.46 0.45 0.41
Avoid walking 0.32** 0.10 0.00
Difficulty bending 0.29** 0.06 0.00
Difficulty carrying 0.26* 0.09 0.03
Transport not used 0.17 0.18 0.05
Difficulty with stairs 0.16 0.07 0.02
Other physical limitation 0.14 0.03 0.00
Drove vehicle 0.06 0.09 0.17
Wheelchair controlled by other 0.01 0.00 0.00
Used wheelchair 0.01 0.00 0.00
Limp or walking aids 0.01 0.00 0.00
Social–activity
Affect school/work 0.51** 0.21** 0.00
Affect personal life 0.45** 0.28** 0.00
Change plans 0.33** 0.27** 0.00
early_acute (n=65)
late_acute (n=65)
convalescent (n=59)
Proportion
0.8
0.6
0.4
0.2
0.0
