# Supplementary material for: Measuring health related quality of life for dengue patients in Iquitos, Peru
Source: PLoS Negl Trop Dis. 2020 Jul 28;14(7):e0008477. doi: 10.1371/journal.pntd.0008477 (PMC7413550; doi:10.1371/journal.pntd.0008477)
Supplement: S2 Fig — All participants from clinic recruitment mode regardless of the number of forms completed. (PDF) [file pntd.0008477.s007.pdf]

S2 Figure

Heatmaps: Proportion of participants reporting symptoms or effects by illness phase (includes all questions)

Figure S2: **All participants from clinic recruitment mode regardless of the number of forms completed** (left column: early-acute, middle column: late-acute, right column: convalescent phase). The proportion is represented by the redness of the cell and the actual proportion is shown by the number in each cell. * Fisher's exact test: $P<0.05$, ** Fisher's exact test: $P<0.01$.

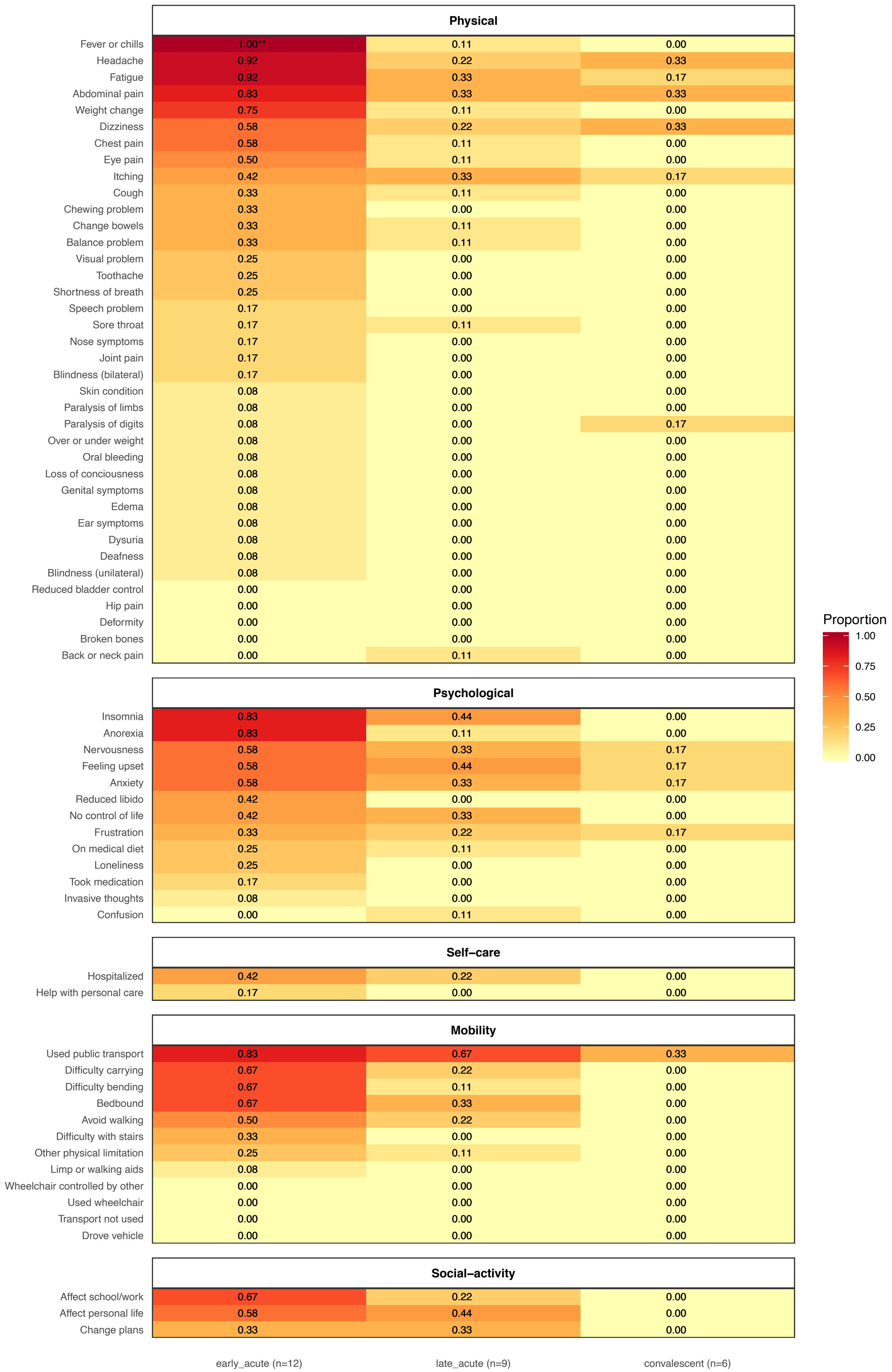
Physical
Fever or chills 1.00** 0.11 0.00
Headache 0.92 0.22 0.33
Fatigue 0.92 0.33 0.17
Abdominal pain 0.83 0.33 0.33
Weight change 0.75 0.11 0.00
Dizziness 0.58 0.22 0.33
Chest pain 0.58 0.11 0.00
Eye pain 0.50 0.11 0.00
Itching 0.42 0.33 0.17
Cough 0.33 0.11 0.00
Chewing problem 0.33 0.00 0.00
Change bowels 0.33 0.11 0.00
Balance problem 0.33 0.11 0.00
Visual problem 0.25 0.00 0.00
Toothache 0.25 0.00 0.00
Shortness of breath 0.25 0.00 0.00
Speech problem 0.17 0.00 0.00
Sore throat 0.17 0.11 0.00
Nose symptoms 0.17 0.00 0.00
Joint pain 0.17 0.00 0.00
Blindness (bilateral) 0.17 0.00 0.00
Skin condition 0.08 0.00 0.00
Paralysis of limbs 0.08 0.00 0.00
Paralysis of digits 0.08 0.00 0.17
Over or under weight 0.08 0.00 0.00
Oral bleeding 0.08 0.00 0.00
Loss of conciousness 0.08 0.00 0.00
Genital symptoms 0.08 0.00 0.00
Edema 0.08 0.00 0.00
Ear symptoms 0.08 0.00 0.00
Dysuria 0.08 0.00 0.00
Deafness 0.08 0.00 0.00
Blindness (unilateral) 0.08 0.00 0.00
Reduced bladder control 0.00 0.00 0.00
Hip pain 0.00 0.00 0.00
Deformity 0.00 0.00 0.00
Broken bones 0.00 0.00 0.00
Back or neck pain 0.00 0.11 0.00
Psychological
Insomnia 0.83 0.44 0.00
Anorexia 0.83 0.11 0.00
Nervousness 0.58 0.33 0.17
Feeling upset 0.58 0.44 0.17
Anxiety 0.58 0.33 0.17
Reduced libido 0.42 0.00 0.00
No control of life 0.42 0.33 0.00
Frustration 0.33 0.22 0.17
On medical diet 0.25 0.11 0.00
Loneliness 0.25 0.00 0.00
Took medication 0.17 0.00 0.00
Invasive thoughts 0.08 0.00 0.00
Confusion 0.00 0.11 0.00
Self–care
Hospitalized 0.42 0.22 0.00
Help with personal care 0.17 0.00 0.00
Mobility
Used public transport 0.83 0.67 0.33
Difficulty carrying 0.67 0.22 0.00
Difficulty bending 0.67 0.11 0.00
Bedbound 0.67 0.33 0.00
Avoid walking 0.50 0.22 0.00
Difficulty with stairs 0.33 0.00 0.00
Other physical limitation 0.25 0.11 0.00
Limp or walking aids 0.08 0.00 0.00
Wheelchair controlled by other 0.00 0.00 0.00
Used wheelchair 0.00 0.00 0.00
Transport not used 0.00 0.00 0.00
Drove vehicle 0.00 0.00 0.00
Social–activity
Affect school/work 0.67 0.22 0.00
Affect personal life 0.58 0.44 0.00
Change plans 0.33 0.33 0.00
early_acute (n=12)
late_acute (n=9)
convalescent (n=6)
Proportion
1.00
0.75
0.50
0.25
0.00
