# Supplementary material for: Measuring health related quality of life for dengue patients in Iquitos, Peru
Source: PLoS Negl Trop Dis. 2020 Jul 28;14(7):e0008477. doi: 10.1371/journal.pntd.0008477 (PMC7413550; doi:10.1371/journal.pntd.0008477)
Supplement: S3 Fig — All participants from community recruitment mode regardless of the number of forms completed. (PDF) [file pntd.0008477.s008.pdf]

S3 Figure

Heatmaps: Proportion of participants reporting symptoms or effects by illness phase (includes all questions)

Figure S3: **All participants from community recruitment mode regardless of the number of forms completed** (left column: early-acute, middle column: late-acute, right column: convalescent phase). The proportion is represented by the redness of the cell and the actual proportion is shown by the number in each cell. * Fisher's exact test: *P<0.05*, ** Fisher's exact test: *P<0.01.*

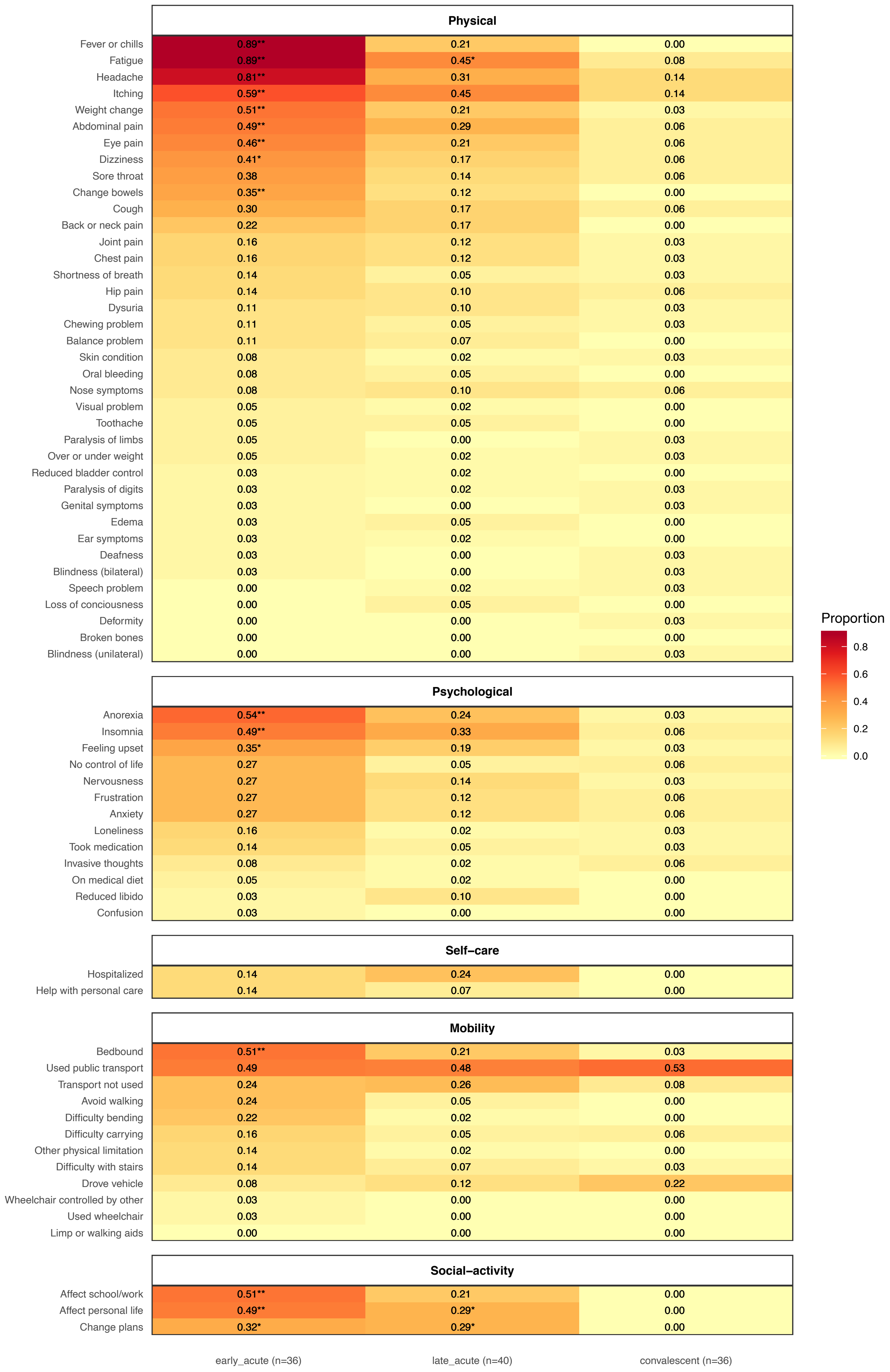
Physical
Fever or chills 0.89** 0.21 0.00
Fatigue 0.89** 0.45* 0.08
Headache 0.81** 0.31 0.14
Itching 0.59** 0.45 0.14
Weight change 0.51** 0.21 0.03
Abdominal pain 0.49** 0.29 0.06
Eye pain 0.46** 0.21 0.06
Dizziness 0.41* 0.17 0.06
Sore throat 0.38 0.14 0.06
Change bowels 0.35** 0.12 0.00
Cough 0.30 0.17 0.06
Back or neck pain 0.22 0.17 0.00
Joint pain 0.16 0.12 0.03
Chest pain 0.16 0.12 0.03
Shortness of breath 0.14 0.05 0.03
Hip pain 0.14 0.10 0.06
Dysuria 0.11 0.10 0.03
Chewing problem 0.11 0.05 0.03
Balance problem 0.11 0.07 0.00
Skin condition 0.08 0.02 0.03
Oral bleeding 0.08 0.05 0.00
Nose symptoms 0.08 0.10 0.06
Visual problem 0.05 0.02 0.00
Toothache 0.05 0.05 0.00
Paralysis of limbs 0.05 0.00 0.03
Over or under weight 0.05 0.02 0.03
Reduced bladder control 0.03 0.02 0.00
Paralysis of digits 0.03 0.02 0.03
Genital symptoms 0.03 0.00 0.03
Edema 0.03 0.05 0.00
Ear symptoms 0.03 0.02 0.00
Deafness 0.03 0.00 0.03
Blindness (bilateral) 0.03 0.00 0.03
Speech problem 0.00 0.02 0.03
Loss of conciousness 0.00 0.05 0.00
Deformity 0.00 0.00 0.03
Broken bones 0.00 0.00 0.00
Blindness (unilateral) 0.00 0.00 0.03
Psychological
Anorexia 0.54** 0.24 0.03
Insomnia 0.49** 0.33 0.06
Feeling upset 0.35* 0.19 0.03
No control of life 0.27 0.05 0.06
Nervousness 0.27 0.14 0.03
Frustration 0.27 0.12 0.06
Anxiety 0.27 0.12 0.06
Loneliness 0.16 0.02 0.03
Took medication 0.14 0.05 0.03
Invasive thoughts 0.08 0.02 0.06
On medical diet 0.05 0.02 0.00
Reduced libido 0.03 0.10 0.00
Confusion 0.03 0.00 0.00
Self–care
Hospitalized 0.14 0.24 0.00
Help with personal care 0.14 0.07 0.00
Mobility
Bedbound 0.51** 0.21 0.03
Used public transport 0.49 0.48 0.53
Transport not used 0.24 0.26 0.08
Avoid walking 0.24 0.05 0.00
Difficulty bending 0.22 0.02 0.00
Difficulty carrying 0.16 0.05 0.06
Other physical limitation 0.14 0.02 0.00
Difficulty with stairs 0.14 0.07 0.03
Drove vehicle 0.08 0.12 0.22
Wheelchair controlled by other 0.03 0.00 0.00
Used wheelchair 0.03 0.00 0.00
Limp or walking aids 0.00 0.00 0.00
Social–activity
Affect school/work 0.51** 0.21 0.00
Affect personal life 0.49** 0.29* 0.00
Change plans 0.32* 0.29* 0.00
early_acute (n=36)
late_acute (n=40)
convalescent (n=36)
Proportion
0.8
0.6
0.4
0.2
0.0
