# Supplementary material for: Measuring health related quality of life for dengue patients in Iquitos, Peru
Source: PLoS Negl Trop Dis. 2020 Jul 28;14(7):e0008477. doi: 10.1371/journal.pntd.0008477 (PMC7413550; doi:10.1371/journal.pntd.0008477)
Supplement: S4 Fig — All participants from contact-cluster recruitment mode regardless of the number of forms completed. (PDF) [file pntd.0008477.s009.pdf]

<u>S4 Figure</u>

Heatmaps: Proportion of participants reporting symptoms or effects by illness phase (includes all questions)

Figure S4: **All participants from contact-cluster recruitment mode regardless of the number of forms completed** (left column: early-acute, middle column: late-acute, right column: convalescent phase). The proportion is represented by the redness of the cell and the actual proportion is shown by the number in each cell. * Fisher's exact test: $P<0.05$, ** Fisher's exact test: $P<0.01$.

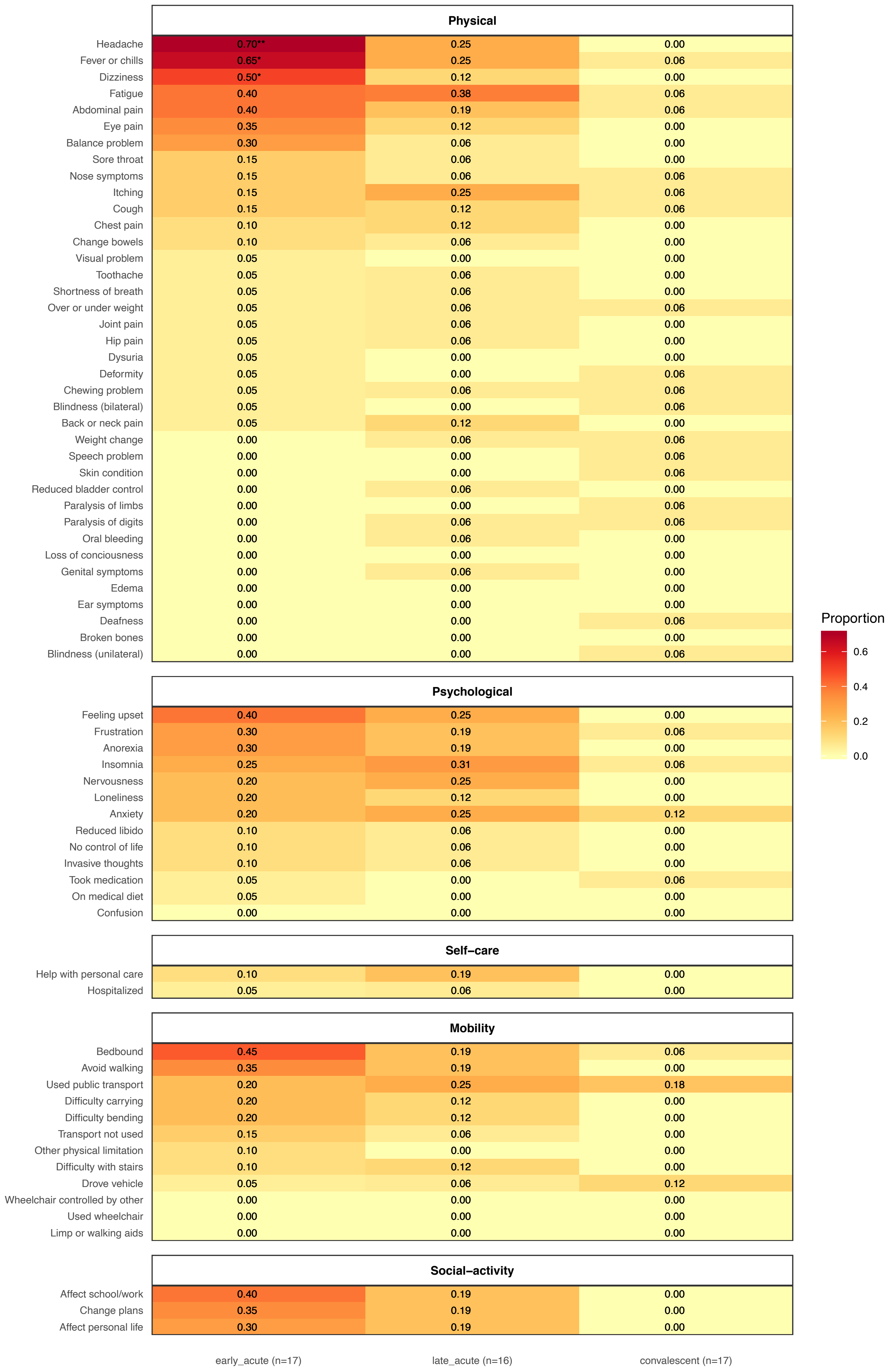

Physical
Headache 0.70** 0.25 0.00
Fever or chills 0.65* 0.25 0.06
Dizziness 0.50* 0.12 0.00
Fatigue 0.40 0.38 0.06
Abdominal pain 0.40 0.19 0.06
Eye pain 0.35 0.12 0.00
Balance problem 0.30 0.06 0.00
Sore throat 0.15 0.06 0.00
Nose symptoms 0.15 0.06 0.06
Itching 0.15 0.25 0.06
Cough 0.15 0.12 0.06
Chest pain 0.10 0.12 0.00
Change bowels 0.10 0.06 0.00
Visual problem 0.05 0.00 0.00
Toothache 0.05 0.06 0.00
Shortness of breath 0.05 0.06 0.00
Over or under weight 0.05 0.06 0.06
Joint pain 0.05 0.06 0.00
Hip pain 0.05 0.06 0.00
Dysuria 0.05 0.00 0.00
Deformity 0.05 0.00 0.06
Chewing problem 0.05 0.06 0.06
Blindness (bilateral) 0.05 0.00 0.06
Back or neck pain 0.05 0.12 0.00
Weight change 0.00 0.06 0.06
Speech problem 0.00 0.00 0.06
Skin condition 0.00 0.00 0.06
Reduced bladder control 0.00 0.06 0.00
Paralysis of limbs 0.00 0.00 0.06
Paralysis of digits 0.00 0.06 0.06
Oral bleeding 0.00 0.06 0.00
Loss of conciousness 0.00 0.00 0.00
Genital symptoms 0.00 0.06 0.00
Edema 0.00 0.00 0.00
Ear symptoms 0.00 0.00 0.00
Deafness 0.00 0.00 0.06
Broken bones 0.00 0.00 0.00
Blindness (unilateral) 0.00 0.00 0.06
Psychological
Feeling upset 0.40 0.25 0.00
Frustration 0.30 0.19 0.06
Anorexia 0.30 0.19 0.00
Insomnia 0.25 0.31 0.06
Nervousness 0.20 0.25 0.00
Loneliness 0.20 0.12 0.00
Anxiety 0.20 0.25 0.12
Reduced libido 0.10 0.06 0.00
No control of life 0.10 0.06 0.00
Invasive thoughts 0.10 0.06 0.00
Took medication 0.05 0.00 0.06
On medical diet 0.05 0.00 0.00
Confusion 0.00 0.00 0.00
Self–care
Help with personal care 0.10 0.19 0.00
Hospitalized 0.05 0.06 0.00
Mobility
Bedbound 0.45 0.19 0.06
Avoid walking 0.35 0.19 0.00
Used public transport 0.20 0.25 0.18
Difficulty carrying 0.20 0.12 0.00
Difficulty bending 0.20 0.12 0.00
Transport not used 0.15 0.06 0.00
Other physical limitation 0.10 0.00 0.00
Difficulty with stairs 0.10 0.12 0.00
Drove vehicle 0.05 0.06 0.12
Wheelchair controlled by other 0.00 0.00 0.00
Used wheelchair 0.00 0.00 0.00
Limp or walking aids 0.00 0.00 0.00
Social–activity
Affect school/work 0.40 0.19 0.00
Change plans 0.35 0.19 0.00
Affect personal life 0.30 0.19 0.00
early_acute (n=17)
late_acute (n=16)
convalescent (n=17)
Proportion
0.6
0.4
0.2
0.0
