# Supplementary material for: Measuring health related quality of life for dengue patients in Iquitos, Peru
Source: PLoS Negl Trop Dis. 2020 Jul 28;14(7):e0008477. doi: 10.1371/journal.pntd.0008477 (PMC7413550; doi:10.1371/journal.pntd.0008477)
Supplement: S5 Fig — Participants from all recruitment modes from subset of individuals who completed a survey in each illness phase. (PDF) [file pntd.0008477.s010.pdf]

<u>S5 Figure</u>

Heatmaps: Proportion of participants reporting symptoms or effects by illness phase (includes all questions)

Figure S5: **Participants from all recruitment modes from subset of individuals who completed a survey in each illness phase** (left column: early-acute, middle column: late-acute, right column: convalescent phase). The proportion is represented by the redness of the cell and the actual proportion is shown by the number in each cell. * McNemar's test: *P<0.05*, ** * McNemar's test: *P<0.01.*

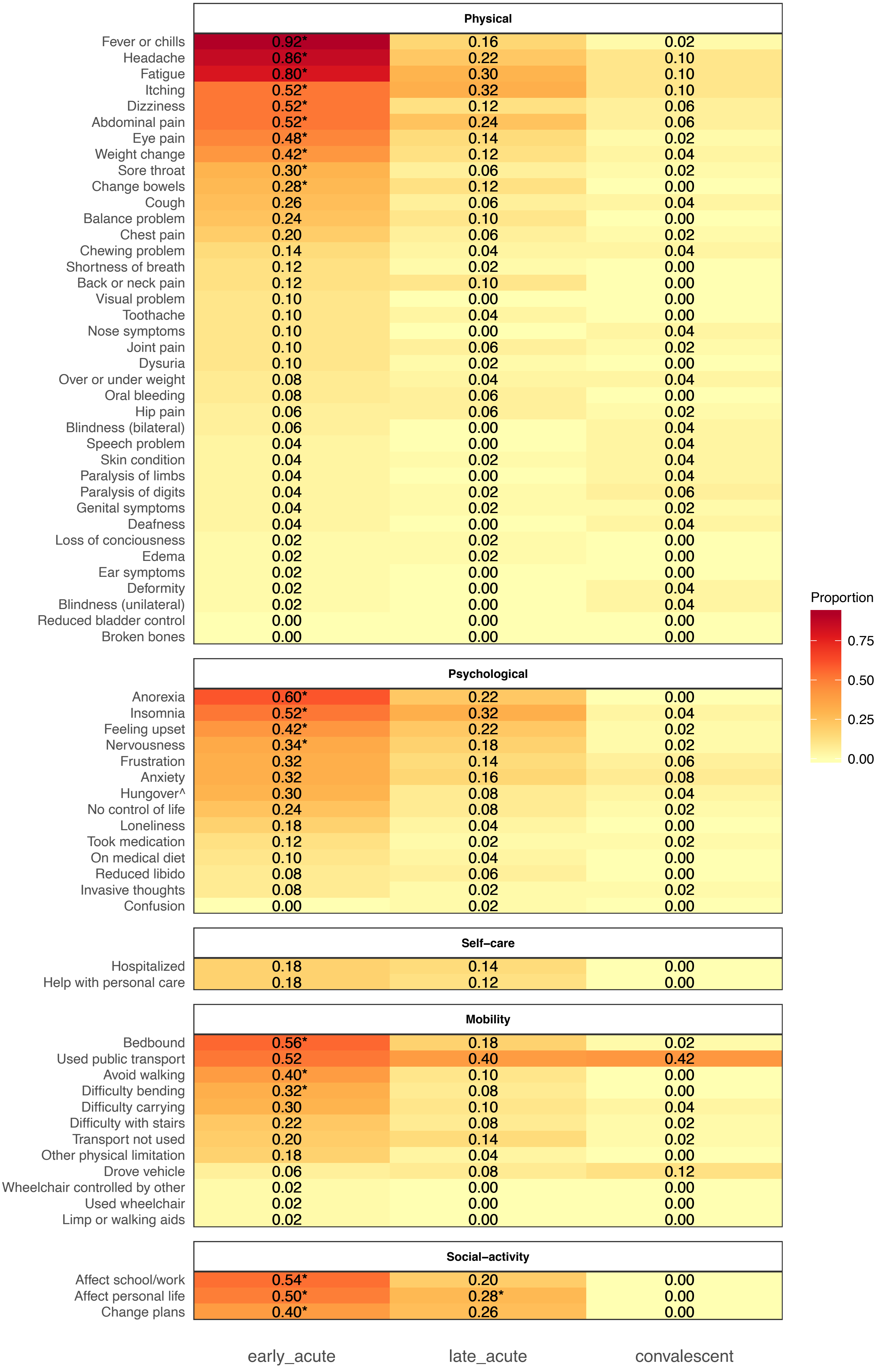
Physical
Fever or chills 0.92* 0.16 0.02
Headache 0.86* 0.22 0.10
Fatigue 0.80* 0.30 0.10
Itching 0.52* 0.32 0.10
Dizziness 0.52* 0.12 0.06
Abdominal pain 0.52* 0.24 0.06
Eye pain 0.48* 0.14 0.02
Weight change 0.42* 0.12 0.04
Sore throat 0.30* 0.06 0.02
Change bowels 0.28* 0.12 0.00
Cough 0.26 0.06 0.04
Balance problem 0.24 0.10 0.00
Chest pain 0.20 0.06 0.02
Chewing problem 0.14 0.04 0.04
Shortness of breath 0.12 0.02 0.00
Back or neck pain 0.12 0.10 0.00
Visual problem 0.10 0.00 0.00
Toothache 0.10 0.04 0.00
Nose symptoms 0.10 0.00 0.04
Joint pain 0.10 0.06 0.02
Dysuria 0.10 0.02 0.00
Over or under weight 0.08 0.04 0.04
Oral bleeding 0.08 0.06 0.00
Hip pain 0.06 0.06 0.02
Blindness (bilateral) 0.06 0.00 0.04
Speech problem 0.04 0.00 0.04
Skin condition 0.04 0.02 0.04
Paralysis of limbs 0.04 0.00 0.04
Paralysis of digits 0.04 0.02 0.06
Genital symptoms 0.04 0.02 0.02
Deafness 0.04 0.00 0.04
Loss of conciousness 0.02 0.02 0.00
Edema 0.02 0.02 0.00
Ear symptoms 0.02 0.00 0.00
Deformity 0.02 0.00 0.04
Blindness (unilateral) 0.02 0.00 0.04
Reduced bladder control 0.00 0.00 0.00
Broken bones 0.00 0.00 0.00
Psychological
Anorexia 0.60* 0.22 0.00
Insomnia 0.52* 0.32 0.04
Feeling upset 0.42* 0.22 0.02
Nervousness 0.34* 0.18 0.02
Frustration 0.32 0.14 0.06
Anxiety 0.32 0.16 0.08
Hungover^ 0.30 0.08 0.04
No control of life 0.24 0.08 0.02
Loneliness 0.18 0.04 0.00
Took medication 0.12 0.02 0.02
On medical diet 0.10 0.04 0.00
Reduced libido 0.08 0.06 0.00
Invasive thoughts 0.08 0.02 0.02
Confusion 0.00 0.02 0.00
Self–care
Hospitalized 0.18 0.14 0.00
Help with personal care 0.18 0.12 0.00
Mobility
Bedbound 0.56* 0.18 0.02
Used public transport 0.52 0.40 0.42
Avoid walking 0.40* 0.10 0.00
Difficulty bending 0.32* 0.08 0.00
Difficulty carrying 0.30 0.10 0.04
Difficulty with stairs 0.22 0.08 0.02
Transport not used 0.20 0.14 0.02
Other physical limitation 0.18 0.04 0.00
Drove vehicle 0.06 0.08 0.12
Wheelchair controlled by other 0.02 0.00 0.00
Used wheelchair 0.02 0.00 0.00
Limp or walking aids 0.02 0.00 0.00
Social–activity
Affect school/work 0.54* 0.20 0.00
Affect personal life 0.50* 0.28* 0.00
Change plans 0.40* 0.26 0.00
early_acute
late_acute
convalescent
Proportion
0.75
0.50
0.25
0.00
