# Supplementary material for: Measuring health related quality of life for dengue patients in Iquitos, Peru
Source: PLoS Negl Trop Dis. 2020 Jul 28;14(7):e0008477. doi: 10.1371/journal.pntd.0008477 (PMC7413550; doi:10.1371/journal.pntd.0008477)
Supplement: S6 Fig — (PDF) [file pntd.0008477.s011.pdf]

S6 Figure

HRQoL score by illness phase

Box and whisker plots demonstrate the distribution of HRQoL scores in each illness phase, dark horizontal line = median, upper limit of box = 75th percentile, lower limit box = 25th percentile, upper whisker extends to the largest value <= 1.5 multiplied IQR, lower whisker extends to the smallest value >= 1.5 x IQR. Red dots represent individual scores. They are partially transparent and therefore appear darker where multiple points overlay each other.

Left hand column **(A, C, E)** shows analyses of all participants regardless of the number of forms completed. **A**: All participants regardless of recruitment mode * Wilcoxon: *P<0.05,* ** Wilcoxon: *P<0.01*. **C:** Comparing recruitment modes within each illness phase * Wilcoxon: *P<0.05,* ** Wilcoxon: *P<0.01*. **E**: Comparing illness phases within each recruitment mode * Wilcoxon: *P<0.05,* ** Wilcoxon: *P<0.01*.

Right hand column **(B, D, F).** This shows analysis of only participants who completed a single form completed in each illness phase. **B**: All participants regardless of recruitment mode * Wilcoxon: *P<0.05,* ** Wilcoxon: *P<0.01*. **D:** Comparing recruitment modes within each illness phase * Wilcoxon: *P<0.05,* ** Wilcoxon: *P<0.01*. **F**: Comparing illness phases within each recruitment mode * Wilcoxon: *P<0.05,* ** Wilcoxon: *P<0.01*.

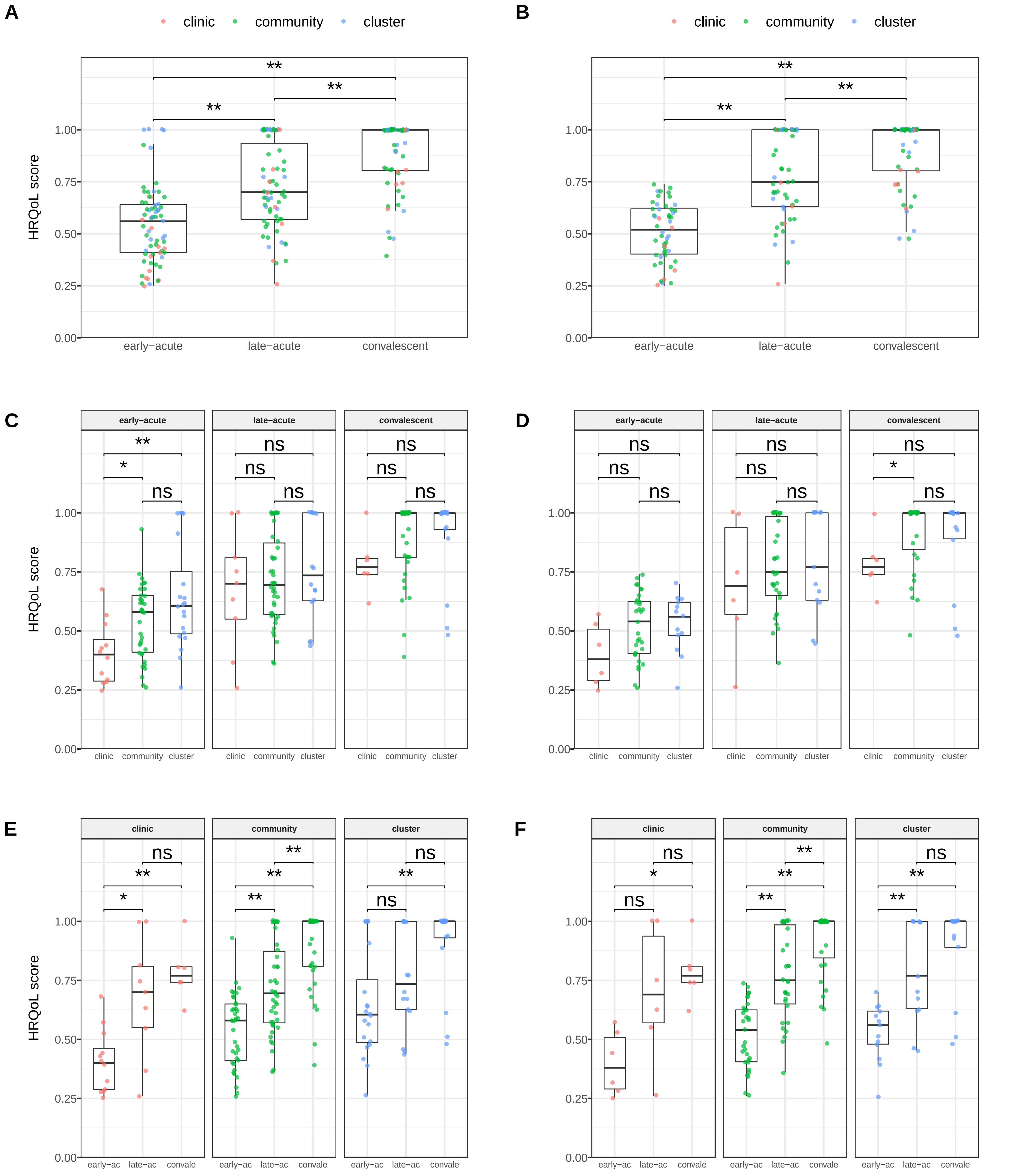

A
B
C
D
E
F
clinic
community
cluster
HRQoL score
early−acute
late−acute
convalescent
early−ac
late−ac
convale
